# Extended reality in cardiovascular care: a systematic review

**DOI:** 10.1093/ehjdh/ztaf070

**Published:** 2025-06-19

**Authors:** Dominika Kanschik, Raphael Romano Bruno, Michel E van Genderen, Patrick W Serruys, Tsung-Ying Tsai, Malte Kelm, Christian Jung

**Affiliations:** Medical Faculty, Division of Cardiology, Pulmonology, and Vascular Medicine, University Hospital Düsseldorf, Heinrich-Heine-University, Moorenstrasse 5, Duesseldorf 40225, Germany; Medical Faculty, Division of Cardiology, Pulmonology, and Vascular Medicine, University Hospital Düsseldorf, Heinrich-Heine-University, Moorenstrasse 5, Duesseldorf 40225, Germany; Department of Adult Intensive Care, Erasmus MC, University Medical Center Rotterdam, Rotterdam, The Netherlands; CORRIB Research Centre for Advanced Imaging and Core Laboratory, University of Galway, Galway, Ireland; CORRIB Research Centre for Advanced Imaging and Core Laboratory, University of Galway, Galway, Ireland; Medical Faculty, Division of Cardiology, Pulmonology, and Vascular Medicine, University Hospital Düsseldorf, Heinrich-Heine-University, Moorenstrasse 5, Duesseldorf 40225, Germany; Cardiovascular Research Institute Duesseldorf (CARID), Moorenstrasse 5, Duesseldorf 40225, Germany; Medical Faculty, Division of Cardiology, Pulmonology, and Vascular Medicine, University Hospital Düsseldorf, Heinrich-Heine-University, Moorenstrasse 5, Duesseldorf 40225, Germany; Cardiovascular Research Institute Duesseldorf (CARID), Moorenstrasse 5, Duesseldorf 40225, Germany

**Keywords:** Augmented reality, Virtual reality, Extended reality, Cardiovascular care, Cardiology, Systematic review

## Abstract

Extended reality (XR) is an emerging technology currently finding its way into various medical fields. This systematic review aimed to compile a comprehensive overview of the current data on XR in cardiovascular medicine. To identify the currently available evidence of the applications of XR in cardiology, we searched PubMed and Web of Science until 31 July 2024 using predefined keywords. After screening, a total of 164 studies were included. Overall, the publications were characterized by very heterogeneous study designs. From the published data, it can already be deduced that XR can support every area of cardiology, from education (*n* = 31) and training (*n* = 36) to peri-procedural care (*n* = 78) and rehabilitation (*n* = 16). Extended reality offers a wide range of applications, and the aim of using these technologies is to optimize the clinical practice. However, these technologies are still in development, and randomized controlled trials are urgently needed to identify their benefits and limitations.

## Graphical Abstract

**Graphical Abstract:**
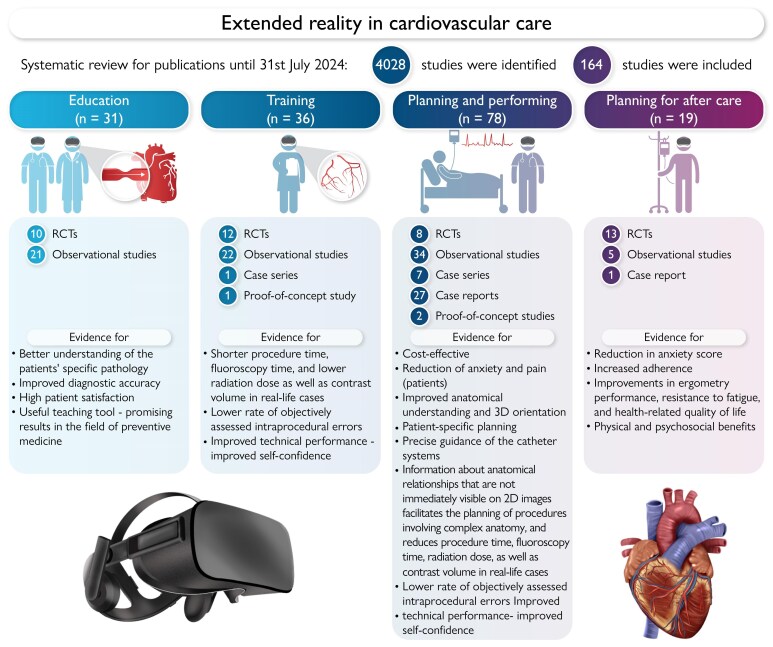

## Introduction

Extended reality (XR), an umbrella term of virtual reality (VR), augmented reality (AR), and mixed reality (MR) has the potential to broadly reshape the field of cardiology by offering innovative approaches to various aspects of patient care and medical practice.^1^ Virtual reality provides the user with a three-dimensional (3D) immersion experience through a headset that covers the entire field of view and controllers that allow interaction with the virtual environment. With AR, virtual elements can be added to a real-world environment by wearing transparent glasses.^2^ Mixed reality combines AR and VR in real time, offering users high-fidelity imaging and retained spatial awareness.^3^ The integration of XR systems into cardiac care opens up new opportunities to improve education, training, pre-procedural planning, guidance of interventions, and ultimately patient outcomes. These technologies provide immersive simulations for medical professionals, allowing them to practice complex procedures such as coronary angiography^4^ or the performance of transseptal puncture in a controlled environment.^5^ This is not only safe but also cost-effective when compared with practical training. Furthermore, XR enables a 3D visualization of cardiac and extracardiac structures, leading to a better understanding of patient morphology and optimizing clinical workflow.^6^ Currently, echocardiographic or computed tomography (CT) data are displayed on two-dimensional screens, which can lead to limitations in the precise evaluation of the findings. High-quality imaging with an exact representation of the individual anatomy and pathology can optimize the performance of procedures and help patients better understand their disease.^7^ Moreover, these technologies are increasingly being used to supplement rehabilitation measures to accelerate the restoration of patients’ quality of life.^8^ Extended reality offers a wide range of applications in cardiology, for students, nurses, doctors, patients, and relatives, which means that the number of publications is continuously increasing but an up-to-date overview is missing.

Therefore, in this systematic review, we present an overview of the current applications of XR in cardiology. We have compiled a summary of the current state-of-the-art applications based on a literature review.

## Methods

### Data sources and searches

Two investigators (D.K. and R.R.B.) systematically searched PubMed and Web of Science databases for publications until 31 July 2024 to identify all published studies reporting on the potential application of XR in cardiovascular medicine. An independent third investigator (C.J.) was involved in the case of discrepancies in the extraction and assessment of the data. The search terms included different combinations of the following MeSH terms: virtual reality, augmented reality, mixed reality, cardiology, structural heart disease, valvular heart disease, congenital heart disease, cardiac catheterization, electrophysiology, heart failure, and cardiac rehabilitation, as well as further terms: XR, interventional cardiology, and device therapy (see Supplementary material online, *Tables S1* and *S2* in the Supplementary material).

### Eligibility and selection criteria

The following articles were eligible: randomized controlled trials (RCTs), prospective or retrospective observational studies (cases and controls, cohort, and cross-sectional studies), and case series or reports. All studies that met the following criteria were included: (i) language: studies published in peer-reviewed journals in English or German; (ii) type of participants: healthcare providers in the field of cardiology or patients with cardiovascular disease, both adults and children; and (iii) type of interventions: VR, AR, or MR.

### Data abstraction and quality assessment

The following data were abstracted: author’s name, year of publication, country, study type, sample size, inclusion criteria, healthcare providers or patient characteristics (age, medical background, and treatment), use of AR/VR/MR, timing, frequency, and duration of application, and results. Two reviewers evaluated the study eligibility and study quality. We assessed the quality of the included studies using the Oxford Levels of Evidence.

### Data synthesis

For all forms of XR, a narrative summary of the findings by one of the literature reviewers was presented, together with a table showing the key features of the included studies. The corresponding author takes responsibility for the integrity of the data and the analysis and has full access to all the data. We decided not to register this review on the International Prospective Register of Systematic Reviews (PROSPERO) since PROSPERO only accepts registrations with health-related outcomes which would limit the review on an early phase application. However, this review conformed to the Preferred Reporting Items for Systematic Reviews and Meta-Analysis.

## Results

**Figure 1:**
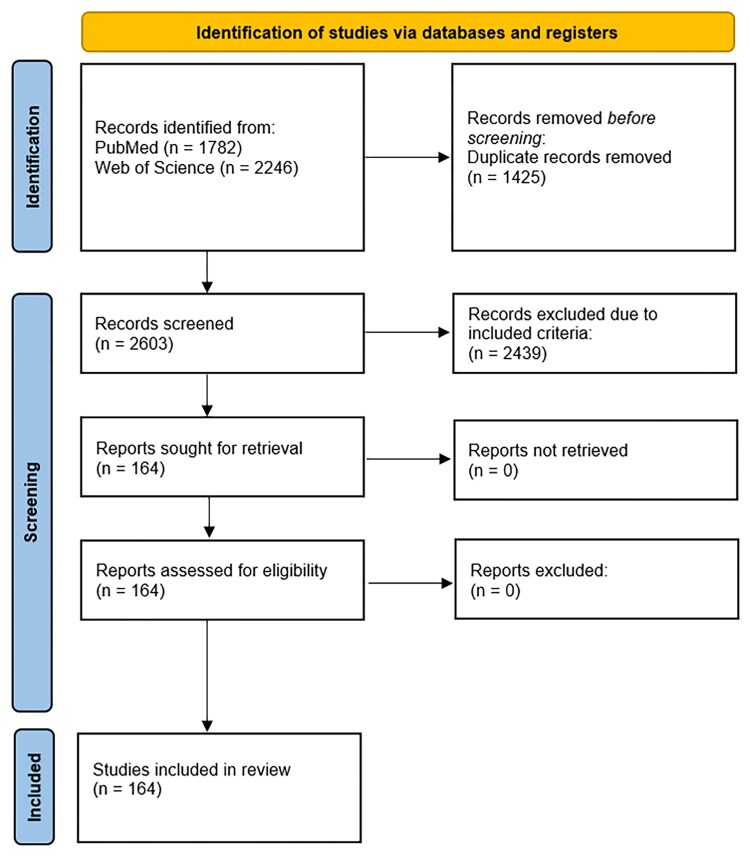
PRISMA 2020 flow diagram for new systematic reviews which included searches of databases and registers only.

After screening with the relevant search terms, 4028 studies were identified. After excluding unsuitable hits (abstract, not German/English, study protocol, review, other subject area, duplicate), a total of 164 studies were included (see *Figure 1*). The use of XR in peri-procedural settings was described in 78 studies, 36 studies evaluated its use for training different cardiovascular procedures, 31 studies dealt with education, and 19 studies concerned cardiac rehabilitation with XR (see *Graphical Abstract*). Supplementary material online, *Table S1* summarizes all studies about the use of XR in cardiology that were included in the search strategy. For each area of cardiology, we discuss different applications, both from the perspective of patients and medical staff.

### Valvular heart disease (*N* = 23)

Valvular heart disease has become increasingly complex and more reliant on advanced imaging guidance. For the diagnosis, treatment, education, and management of valvular heart disease, XR holds significant promise. Imaging data such as CT scans can be used to create patient-specific highly detailed 3D models that allow physicians to visualize a patient’s anatomy and simulate various treatment options.^9^ As a result, the most appropriate interventional approach can be selected and optimal outcomes can be achieved. Ruyra *et al*.^10^ investigated the use of VR for pre-procedural planning of transcatheter aortic valve implantation (TAVI). The study included 11 patients, and in 5 cases, VR use led to a change in implant strategy. Especially in complex scenarios such as a short distance between the bioprosthesis and the coronary ostia or a short mitral-aortic distance, VR’s immersive experience could help create the most personalized treatment plan for each patient. Kamiya *et al*.^11^ evaluated in a study with cast models the accuracy of VR measurements of the aortic valve. The CT data of the solidified cast were processed through VR software and 2D multiplanar reconstruction. All measurements were compared against physical measurements, and it could be shown that VR measurements seem considerably more accurate than the current standard 2D measurements. To save contrast agent and radiation, Currie *et al*.^12^ proposed an alternative guidance system using AR and transoesophageal echocardiography (TEE) to guide TAVI. They showed that by using AR guidance, comparable deployment accuracy could be achieved without the use of contrast agent and radiation. Sadri *et al*.^13^ developed an AR guidance system for positioning cerebral embolic protection devices that reduced the need for additional intra-procedural angiograms before device placement with no significant difference in intervention time and improved subjective performance as well.

Innovative technologies have also been found to be applicable in the field of mitral valve interventions. The neo-left ventricular outflow tract obstruction is one of the most feared complications, causing haemodynamic compromise, and is a predictor of 1-year mortality, which requires precise pre-procedural planning.^14^ In two case reports, it was shown that the use of VR can significantly facilitate the planning of percutaneous valve-in-valve transcatheter mitral replacement by providing a detailed visualization of the 3D spatial relationship between the implants and the surrounding anatomy.^15,16^

The modalities can also be used from the patient’s perspective. As patients often complain of pain and anxiety during procedures such as TAVI, Bruno *et al*.^17^ investigated whether VR application can alleviate these symptoms. In an RCT with 32 patients, they demonstrated that the use of VR interventions during TAVI was safe and feasible and led to a reduction in pain and anxiety. These results were confirmed in a further trial with 207 patients.^18^

The application of XR to valvular heart disease can significantly enhance diagnosis, treatment, and patient management. While proof-of-concept studies exist, there are limited data on the efficacy and reproducibility of VR-/AR-assisted navigation in live interventions. The integration of XR with real-time imaging modalities such as fluoroscopy or intraoperative echocardiography is still in its early stages. There is no consensus on the standardization of XR technologies for SHD procedures and no established regulatory framework for their widespread clinical adoption. Furthermore, XR-based simulators have been created for training healthcare providers in various cardiology procedures, but these are still relatively limited in scope when it comes to complex SHD interventions. Future advancements in XR tools and personalized therapy will further transform the care of these patients, improving both procedural success and long-term outcomes.

### Non-valvular structural heart disease (*N* = 12)

By enhancing visualization, improving procedural precision, and offering immersive training and education, XR holds the potential to improve both patient outcomes and healthcare provider expertise in the field of structural heart disease.^19^ Left atrial appendage occluder implantations are complex and highly variable procedures due to the complex and highly variable 3D anatomy of the left atrial appendage.^20^ Heidari *et al*.^21^ evaluated in an observational study with 21 patients the feasibility, accuracy, and reproducibility of visualizing the LAA in VR for pre-procedural planning. They demonstrated that VR allows precise and reproducible measurements with enhanced 3D orientation, which can make planning and thus device selection easier. Sawada *et al*.^22^ demonstrated the application of a VR simulator for the detection of an ideal delivery sheath curve for a patient with complex anatomy, and Zbroński *et al*.^23^ took the next step and investigated both the pre- and intra-procedural application of AR for LAAC. The 3D reconstruction has proven to be a useful adjunct to the assessment of the LAA and neighbouring structures.

In patients with paravalvular leakage (PVL) and high operative risk, a transcatheter PVL closure can be an effective treatment option.^24^ Due to the highly patient-specific nature of PVL dimensions and locations, detailed pre-operative planning is also required here. Sadeghi *et al*.^25^ investigated a multimodal approach consisting of TEE, CT, 3D computational model, and VR in six cases. Several advantages of the 3D modalities application have been demonstrated. Especially due to the detailed representation of 3D geometry and their in-depth perception, these techniques can complement conventional methods.

There are significant opportunities to advance the application of XR technologies in non-valvular structural heart disease. Extended reality–based simulation and training systems have been developed for general cardiology procedures, but there is limited evidence of high-fidelity simulation platforms specifically focused on non-valvular structural heart diseases. The clinicians may not have access to sufficient, realistic simulation environments to practice these procedures. Furthermore, XR has shown potential in educating patients about their heart conditions, particularly in areas like ASD and LAA. However, the use of XR for engaging and educating patients on these conditions remains limited. By focusing on developing XR solutions for pre-procedural planning, real-time intra-procedural navigation, training, patient education, and outcome evaluation, researchers could help improve the quality of care and clinical outcomes for patients with these conditions. The development of standardized XR protocols and validation studies would be crucial in ensuring the effective integration of these tools into clinical practice.

### Congenital heart diseases (*N* = 28)

With numerous applications across the spectrum of patient care, XR is becoming an increasingly valuable tool for the education and management of congenital heart diseases (CHDs). In terms of effective education, using VR students and medical staff can gain a deeper understanding of CHD by participating in immersive, interactive, and practical learning experiences. d’Aiello *et al*.^26^ evaluated in a study with 59 medical students the potential impact of MR technology on medical training in CHD. They were randomly allocated into three groups: the first group attended a lecture in which traditional slides were projected onto a flat screen, the second group was shown slides incorporating videos of holographic anatomical models, and the third group wore immersive, head-mounted devices to interact directly with holographic anatomical models. All students were able to describe the anatomy of the taught defect (sinus venosus atrial septal defect and the superior vena cava type with partial anomalous pulmonary venous return), but only the students interacting with the holograms were able to answer the questions about planning, percutaneous treatment with stenting, and potential complications. In another study, 27 paediatric residents and 3 nurse practitioners explored models of a developmentally typical heart and tetralogy of Fallot pathology.^27^ Participants reported understanding VR models better than 3D-printed models or traditional instructional models. Most participants felt comfortable with the technology, and 87% of participants preferred VR over 3D printed models. Patel *et al*.^28^ conducted a prospective, single-blinded, randomized trial to assess the efficacy of using VR to teach about a CHD lesion. The intervention group (24 subjects) used a VR headset to visualize a lecture on the atrioventricular canal with 3D heart models, while the control group (27 subjects) used a desktop computer interface with the same models. Participants in the intervention group reported a better learning experience and self-assessment suggesting VR may increase learner engagement in understanding CHD. However, there was no statistically significant difference in the knowledge acquisition observed between the groups. Lim *et al*.^29^ created a VR curriculum for residents participating in paediatric cardiology rotations. It developed modules to teach six common congenital heart lesions, as well as narrative scripts, and the trainees completed a validated assessment tool with 27 questions. There were 80 trainees in the control group and 52 in the intervention group. Participants in the intervention group achieved higher scores on the assessment, and the analysis showed significant improvement in the intervention group for questions specifically testing visuospatial concepts. All users recommended integrating the programme into residency programmes.

Furthermore, VR can facilitate detailed pre-procedural planning for interventions in CHD. Galeczka *et al*.^30^ presented a case report of a patient with a history of persistent ductus arteriosus and current left pulmonary artery (LPA) stenosis. Through the use of different 3D reconstruction profiles, they could visualize the LPA stenosis from both the outside and the inside of the pulmonary artery. Moreover, a simulation of LPA stenting was performed, and angiography projections were planned. In another case, intra-procedural use of MR holographic display of 3D CT angiography data, featuring sterile touchless control of holographic image shared between the interventional and imaging team, was presented to support percutaneous patent ductus arteriosus.^31^ Topuzov *et al*.^32^ evaluated the application of VR in planning interventional treatment of aortic coarctation. Before the procedure, 20 patients underwent CT scans, which were used to create a virtual 3D model of the aorta. Five stents were identical or very similar, 12 simulations had slight, potentially avoidable errors in stent size or diameter, and 3 cases differed significantly from each other. Overall, in 14 cases, the location of the stent was concordant between the simulation and reality, and in the remaining six cases, the simulated stent was located lower than the actual one. Not only CT images but also cardiac magnetic resonance (CMR) images can be visualized using VR. Ghosh *et al*.^33^ created 3D models from CMR images of a 28-month-old girl with multiple ventricular septal defects. This allowed the defects to be localized precisely, which helped the team to determine the treatment approach.

Summarizing, XR technologies have significant potential to enhance multiple aspects of congenital heart disease management. However, there is still considerable room for development in CHD. More research is needed to develop XR tools that allow for comprehensive, patient-specific 3D visualizations, particularly for rare or complex congenital conditions. Moreover, while there are tools for monitoring adult cardiovascular conditions, there are few XR-based solutions tailored to the lifelong management of patients with congenital heart disease, who may require periodic assessments as they age. Furthermore, coping with congenital heart disease can be emotionally and psychologically taxing. There is a lack of XR applications that provide psychological support, relaxation techniques, or coping strategies for patients and their families. Expanding the use of XR in these areas could greatly improve clinical outcomes, enhance patient and family engagement, and streamline the training of healthcare providers dealing with complex congenital heart conditions. Future research should focus on validating these technologies, improving their clinical integration, and ensuring they provide tangible benefits in terms of patient care and outcomes.

### Cardiac catheterization (*N* = 35)

Through advanced training tools, enhanced pre-procedural planning, and real-time guidance, the XR is revolutionizing cardiac catheterization. Popovic *et al*.^34^ evaluated the effectiveness of VR training in coronary angiography and the transferability of acquired skills to the real world. Compared with the control group, the duration of the procedure in real-life cases was shorter, the radiation dose lower, and the overall score for procedural competence higher after the simulator-based training. Three further studies confirmed the results and suggested that curriculum-based, supervised VR simulation training can improve the performance level of cardiology residents during coronary interventions.^4,35,36^ Positive experience has also been gained in the field of carotid artery angiography. Cates *et al*.^37^ showed in their study that experienced interventional cardiologists trained on the VR simulator performed significantly better than their equally experienced controls and had a significantly lower rate of objectively assessed intraoperative errors in carotid artery angiography. Additionally, VR can be useful for planning complex coronary procedures. Higami *et al*.^38^ showed in a case report of a patient with a chronic total occlusion (CTO) of the right coronary artery, in whom the team had difficulty inserting the catheter during the initial examination, that VR simulation prior to PCI helped overcome the difficulties in guiding the catheter and enabled a successful procedure. Opolski *et al*.^39^ have taken the next step and evaluated AR-assisted percutaneous revascularization of CTO. The cardiologists wore AR glasses during the CTO-PCI to obtain additional information on tortuosity and calcification. The use of AR glasses was feasible and highly rated by the PCI operators. No serious adverse cardiovascular events occurred. Compared with standard CTO-PCI, AR-assisted recanalization of CTO more frequently used the wire of first choice and contrast exposure was lower. However, there were no significant differences between the two groups in terms of success rates and safety outcomes.

These procedures are also often associated with anxiety and pain for patients.^2^ For this reason, Gökçe and Arslan^40^ investigated in an RCT the effects of VR and acupressure interventions on pain, anxiety, vital signs, and comfort during catheter removal. One hundred and fifty-three patients were randomized, with 51 in the VR group, 51 in the acupressure group, and 51 in the control group. Both intervention groups had significantly lower pain and anxiety scores, as well as higher comfort scores, compared with the control group. Similar results were presented by Morgan *et al*.^41^ in the VIRTUAL CATH Trial with 64 patients (33 in the VR arm and 31 in the control arm). The VR group had a significantly greater reduction in anxiety levels from baseline to post procedure than the control group. In addition, the VR group had a better procedural understanding and higher overall satisfaction. However, Larsson *et al*.^42^ demonstrated in a RCT with 156 patients, where the intervention group used a VR mask in the transfer room prior to invasive coronary angiography while the control group performed the procedure as usual, that the pre-operative use of a VR mask for anxiolytic purposes in the context of coronary angiography did not lead to a reduction in anxiety levels.

In summary, the use of XR in the field of cardiac catheterization is still in its early stages. There is a lack of patient-specific, 3D visualizations created using XR that would allow clinicians to explore a detailed and immersive representation of the patient’s coronary anatomy. Moreover, there is a lack of XR technologies that help visualize and assess post-procedural outcomes in real time, such as evaluating the efficacy of stent placement or detecting potential complications like restenosis or vessel dissection. Furthermore, there is little research on how XR technologies can be used to reduce radiation exposure during catheterization procedures. Future research should focus on developing immersive, patient-specific 3D models, real-time navigation systems, AI-assisted decision tools, and interactive training to fill these gaps and improve the accuracy, safety, and efficiency of catheterization procedures.

### General training, patient education, and heart failure (*N* = 19)

Providing detailed insights into cardiac structure and function, echocardiography is an essential tool in cardiology. O’Sullivan *et al*.^43^ evaluated the usefulness of a VR echocardiographic approach in teaching echocardiography to paediatric trainees compared with live demonstration. All 15 participants confirmed that VR echocardiography is a useful teaching tool. Sixty-seven per cent of the subjects rated VR echocardiography as the same or better than live demonstrations. Only one person reported a side effect.

Cardiopulmonary resuscitation (CPR) training is another important skill that everyone should be familiar with and AR has the potential to offer a novel approach in this field. Balian *et al*. evaluated the feasibility of an AR CPR training system for healthcare providers.^44^ The results were in line with the recommendations of the guidelines for high-quality CPR. Participants were overwhelmingly satisfied with the system and 94% were willing to use the application for future CPR training.

As a result of illnesses such as heart failure, patients also experience pain during their hospital stays. Groninger *et al*.^45^ presented a study with 88 participants hospitalized with advanced heart failure. The impact of the VR experience in terms of self-reported pain, quality of life, general distress, and satisfaction was compared with an active control with 2D guided imaging. A significant improvement in pain scores was achieved in both groups, although the reduction in pain scores was greater in the VR group. Both groups did not show a significant change in quality of life or general distress scores.

Patient information after a myocardial infarction (MI) plays an important role in secondary prevention. Therefore, Hilt *et al*.^46^ investigated whether XR medication applications can support patient education about the function of statins after MI. Twenty-two MI patients were enrolled: 12 in the intervention group and 10 in the control group. Eighty-three per cent of patients in the intervention group improved their statin knowledge by using the application. The improvement was primarily due to a better understanding of statin mechanisms and secondary preventive effects.

There are substantial opportunities to integrate XR technologies into general training and heart failure management. However, there is a lack of XR-based educational tools that can visually and interactively demonstrate the pathophysiology of heart failure in an immersive and patient-specific way. There is a lack of XR-based simulators to train clinicians in diagnosing heart failure and recognizing symptoms in diverse clinical settings. The diagnostic challenges, such as distinguishing between heart failure with preserved ejection fraction and heart failure with reduced ejection fraction, are complex and often involve nuanced decision-making. By addressing the gaps in areas like pathophysiology visualization, diagnosis, treatment management, advanced therapies, and patient education, XR can enhance both the learning experience for healthcare professionals and the engagement of patients in their own care. As these technologies evolve, the potential for improving clinical outcomes and empowering both healthcare providers and patients becomes increasingly apparent.

### Electrophysiology (*N* = 19)

The examinations and treatments in electrophysiology (EP) involve complicated procedures that require a thorough understanding of the anatomy and EP of the heart. Virtual reality training can change the way medical professionals learn and practice this complex specialty. A cohort study compared traditional and VR teaching methods for learning 3D cardiac anatomy.^47^ There was considerable interest in including VR learning environments into the EP fellowship curriculum. However, a third of the participants found using the VR system difficult, and its impact on exam performance varied. There was a strong interest among participants in implementing VR into the EP fellowship curriculum. However, one-third of participants found the VR system difficult to use and the impact of the VR session on exam performance varied. De Ponti *et al*.^5^ compared in a RCT (*n* = 14) the performance of fellows in transseptal catheterization after VR and conventional training. The training time was significantly longer in the control group. Furthermore, the post-training performance scores were lower, and the number of recurrent errors was higher. An interesting approach is the intra-procedural application of XR during electrophysiological examinations. Silva *et al*.^48^ developed an Enhanced Electrophysiology Visualization and Interaction System (ĒLVIS) which uses an XR system to display patient-specific data in real-time during minimally invasive EP procedures. System use resulted in few interactions during point navigation to confirm the position of the catheter, which can impact efficiency, workflow, and team dynamics in the EP lab. The physicians found the system comfortable and easy to use. In addition, 93% of participants felt that a 3D display of data with the ability to change the viewing angle was easier to interpret than the current standard, which is displayed on 2D monitors. In another study, it was shown that the implementation of the AR system for navigational mapping did not lead to a prolongation of the procedure.^49^

Educating patients about EP is also crucial to ensuring that they understand their condition, treatment options, and procedures. Two studies showed that VR-assisted pre-procedural patient education provided better information and procedure-related knowledge, increased satisfaction, and reduced procedure-related worries.^50,51^ Roxburgh *et al*.^52^ investigated the feasibility and effectiveness of VR in patients undergoing atrial fibrillation ablation under conscious sedation. A comparison of the VR group and the control group showed that the means of perceived pain, assessed with the visual analogue scale, were lower in the VR group, and the levels of comfort were higher there. However, the consumption of medication was similar between groups. On the other hand, Coulibaly *et al*.^53^ showed that VR use was associated with less comfort during the procedure and that VR had no effect on pain. Here too, medication consumption was the same.

In the field of EP, XR holds considerable promise and XR can improve various aspects of EP, including pre-procedural planning, intra-procedural guidance, training, and patient education. However, a significant gap exists in the use of XR for 3D visualization and simulation of the heart’s electrical pathways and arrhythmogenic substrates before procedures. Furthermore, there is a lack of high-fidelity XR-based training platforms specifically for electrophysiology. Addressing these gaps could significantly enhance the precision and outcomes of electrophysiology procedures and improve patient understanding and engagement in their care. Future studies should focus on developing XR-based tools that provide immersive, data-driven insights for both clinicians and patients, with the potential to revolutionize the field of electrophysiology.

### Cardiac resynchronization therapy devices (*N* = 9)

Virtual reality is making significant strides in the field of device therapy, particularly in cardiology, where it enhances healthcare professional training. Maytin *et al*.^54^ evaluated in an RCT with eight EP fellows the use of a VR lead extraction simulator. In comparison with the control group, the VR group performed better in patient preparation and procedure execution. A simulation complication was experienced by all participants in the control group, compared with one fellow in the VR group. Moreover, there was a tendency towards excess pushing vs. pulling forces in the control group, and the time for lead removal was significantly shorter in the VR group. The potential benefits of 3D visualization were also evaluated intra-procedural. Opolski *et al*.^55^ presented a case report of a patient with congenitally corrected transposition of great arteries and dextrocardia, AR-guided transcatheter pacemaker implantation. The case was complex due to both ventricles being inverted and rotated to the right and trabeculation of the subpulmonic ventricle and interventricular patch limiting identification of the optimal stimulation site. The AR application helped to determine the optimal fluoroscopy angles and control the positioning of the pacemaker. In another case, a successful application of the AR during a cardiac resynchronization therapy (CRT) device implantation was demonstrated.^56^ In addition to enabling a transparent, global view for the operator, the system did not limit the operator’s vision. Drozdova *et al*.^57^ evaluated the use of VR videos on pacemaker implantation for patient education. However, there was only a trend towards higher educational scores in the VR group but no significant differences in the quality of education compared with standard care.

In the field of CRT devices, XR has the potential to enhance multiple aspects of care, from pre-procedural planning to device optimization and patient education. However, there is a lack of XR-based tools for patient-specific, pre-procedural planning for device implantation. While pre-procedural planning is common for procedures such as TAVR, the use of XR for visualizing and simulating device placement for implants such as pacemakers is not widespread. Furthermore, there is a lack of XR systems that provide real-time, augmented guidance during the implantation of cardiac devices. Real-time feedback using XR technologies, such as overlaid 3D models, has not been fully explored. By addressing these gaps, XR can improve the precision, efficiency, and patient-centred care of device therapy, leading to better outcomes for patients. Future research should focus on integrating XR with existing technologies to create more comprehensive, interactive, and effective care pathways for patients.

### Cardiac rehabilitation (*N* = 19)

Extended reality is increasingly being integrated into rehabilitation programmes, offering innovative ways to assist patients in recovering from cardiovascular diseases. As such, Jóźwik *et al*.^58^ evaluated the addition of eight sessions of VR therapy with the VR TierOne device to standard rehabilitation. It is based on the metaphor of a Virtual Therapeutic Garden where the patient can calm down and relax. The Hospital Anxiety and Depression Scale and the general stress level were statistically significantly lower in the intervention group compared with the control group. García-Bravo *et al*.^59^ investigated the benefits of VR training with the XBOX ONE console and the Kinect sensor in patients with ischaemic heart disease. The VR training protocol proposed aerobic activities, such as dodging objects, avoiding obstacles, and imitating postures, squats, and steps. This programme improved ergometry, metabolic equivalents, fatigue resistance, and health-related quality of life with very high adherence and satisfaction. In another trial, it was also shown that the patients had accepted the VR-based training well and were satisfied with it.^60^ In addition, patients reported physical, mental, and social benefits provided by VR training as well as a higher training intensity than with conventional training. Gulick *et al*.^61^ investigated the potential benefits of VR walking trails within cardiac rehabilitation. Seventy-two patients were enrolled, 41 in the intervention group and 31 in the control group. Despite the positive reactions of the patients, no significant differences were found between the intervention and control group in the 6-min walk test. Furthermore, there were not any statistically significant differences between groups in terms of education or satisfaction.

In the field of cardiac rehabilitation, XR has significant potential to enhance both patient engagement and the effectiveness of rehabilitation programmes. Some XR applications have been explored to create generalized virtual exercise environments for patients in cardiac rehabilitation. However, personalized rehabilitation programmes that cater to individual patient needs (based on their specific cardiac conditions, fitness levels, and rehabilitation progress) are still limited. While some devices provide biofeedback (e.g. heart rate or physical activity), integration of XR with continuous monitoring remains underexplored. Areas like personalized rehabilitation, real-time monitoring, psychological support, and long-term cardiovascular health management all present opportunities for further exploration. Bridging these gaps through future research and development could lead to more engaging, effective, and accessible rehabilitation programmes, ultimately improving patient outcomes and adherence.

## Summary

Overall, XR offers many applications in cardiology, including teaching medical students and residents, patient education, pre-procedural planning, intra-procedural use, research, and cardiac rehabilitation. Through an immersive, interactive learning experience, XR is an interesting addition to traditional teaching methods. Especially in the field of CHD, where the anatomy of patients is often very complex, 3D models of the heart allow students to explore the anatomy of the heart and understand the complexity of various defects such as atrial septal defect, ventricular septal defect, tetralogy of Fallot, or double outlet right ventricle.^62^ In training, these technologies enable medical professionals to practice complex procedures such as personalized percutaneous coronary intervention,^63^ carotid artery angiography,^37^ or ablation^64^ in a controlled and safe environment. Compared with standard training, this is cost-effective and can be repeated as often as required.^65^ Consequently, self-confidence and competence can be effectively developed. Through detailed, interactive, and patient-specific visualizations by converting 2D images into 3D interactive formats, XR was also evaluated in the pre-procedural planning of multiple percutaneous interventions. Extended reality can help interventionalists plan complex procedures such as TAVI,^10^ transcatheter mitral valve-in-valve replacement,^16^ LAAC,^21^ or closure of sinus venosus defect^66^ with greater accuracy and certainty. Furthermore, XR simulations may help us determine the appropriate device size, sheath type, and optimal angulation of the deflectable sheath. In addition, these technologies provide real-time intra-procedural guidance, enhanced visualization, and precise navigation, which can reduce patient recovery and improve patient outcomes. The evaluated applications were also highly diverse, being utilized during interventions such as coronary angiography,^39^ pulmonary vein isolation,^67^ cardiac electrophysiological testing,^49^ or cardiac resynchronization device implantation.^56^ With MR, 3D images of the heart can be projected onto the interventional field, allowing catheter-based interventions to be navigated in real time. From the patient’s perspective, these methods can also support every phase of treatment. Extended reality could help patients better understand and visualize their heart conditions, diagnosis, and planned procedure. This can enhance commitment to the treatment plan and increase compliance. Intra-procedural use, for example, during TAVI^17^ or electrophysiological procedures^53^ can minimize anxiety and pain, which can improve the patient’s experience and overall satisfaction with medical care. In the area of prevention, these technologies can help to communicate the importance of taking medication such as statins for coronary heart disease^46^ or anticoagulation for atrial fibrillation.^68^ Furthermore, positive effects of the additional use of XR in the field of cardiac rehabilitation were reported. It led to an increase in activity and was well tolerated by the patient without any adverse events.^69^

Although the application of XR has great potential in cardiology and offers promising effects in many areas, contradictory results have also been documented. Furthermore, there are only a limited number of prospective, randomized trials, making it difficult to perform a meta-analysis or to use methods to assess the risk of bias in the included studies. Due to the large number of observational studies with small sample sizes, case series, and case reports, it is difficult to transfer the results to the general population. Extensive clinical validation is necessary to ensure that XR improves patient outcomes in the real world. Moreover, XR systems with high performance can be expensive, limiting their widespread application in hospitals and clinics. The AR/VR/MR software used was also very different, which can impede the detailed visualization necessary for medical applications. The available XR evidence comes from quite diverse platforms, which limits the reproducibility and generalizability of the data. Moreover, most studies are not blinded, which can influence participants’ behaviour, resulting in high-performance bias. Beyond that, adapting to these new technologies requires intensive training of medical staff, which can hinder their widespread introduction. The VR/AR headsets may cause discomfort or disorientation in some users, limiting their effectiveness and usability. Advances in XR hardware, particularly in lighter, more ergonomic headsets, could improve comfort for users during long procedures. In addition, an enhanced user interface and more intuitive controls could improve XR’s performance. The lack of regulatory approvals is another significant barrier to the adoption and use of XR technologies in clinical practice. The regulatory framework plays a critical role in ensuring that new technologies are safe, reliable, and ethical, especially in health and education fields. Many XR environments collect and process large amounts of data, including personal and biometric information, and the absence of comprehensive data handling, privacy, and security regulations can result in risks of data misuse and breaches.

Overall, due to the lack of possibility to adequately compare the studies, it is currently difficult to make a statement about the exact benefits and limitations of these technologies. The validation of XR in the environment remains a critical issue.

## Outlook

Extended reality technologies can revolutionize how cardiologists diagnose, treat, and educate patients, enhancing clinical outcomes, patient engagement, and procedural efficiency. Supplementary material online, *Table S2* summarizes all registered clinical trials in the XR cardiology field. Projects are exploring how XR can enhance catheter navigation by overlaying real-time imaging data with a patient’s anatomy during complex procedures, such as MitraClip implantation and percutaneous coronary intervention. Furthermore, in many studies, the technologies are used to educate patients about their heart condition in a highly visual and interactive way. In addition to educating patients on planned procedures such as coronary angiography, they can also provide information on diseases such as arterial hypertension and ATTR amyloidosis for preventive health. Moreover, numerous clinical trials are investigating the intra-procedural application of XR to reduce stress and anxiety during interventions such as electrophysiological examination or implantation of cardiac electronic devices.

The purpose of these XR-focused clinical trials is to determine whether XR technology can reduce complications in high-risk cardiovascular interventions and increase procedural accuracy, improve retention of knowledge and skills in medical training, promote shared decision-making, and reduce anxiety as well as stress among patients by improving their understanding of their conditions, and reduce re-hospitalization and improving the quality of life by enhancing patient adherence to cardiac rehabilitation. While the data from these clinical trials are still emerging, early results are promising.

## Future perspectives

The use of XR in cardiology is expected to expand as the technology matures. As software and devices continue to develop, becoming lighter, more cost-effective, and offering higher quality, it can be anticipated that acceptance will increasingly grow among both medical staff and patients. In addition, several randomized controlled trials are currently being conducted to evaluate the efficacy and safety of XR applications. The endpoints in future studies must be clearly defined to better assess the benefits and limitations. It is hoped that the results of these studies will ultimately promote regulatory approval, clinical acceptance, and widespread adoption of XR-based solutions for cardiac treatment. Extended reality signifies a new era in cardiology and can redefine cardiovascular care. The potential applications that could revolutionize patient care, education, diagnostics, and rehabilitation are numerous. Especially in the field of structural heart disease, we expect to make significant progress in the coming years. Improvements in 3D imaging allow interventional cardiologists to simulate a specific patient’s procedures in a highly detailed virtual environment. This allows complex catheter-based interventions to be controlled more precisely, which could reduce errors and improve outcomes.

In the course of technological progress and the increasing use of innovative technologies in medicine, XR can thus establish itself as a cornerstone of cardiology.

## Conclusion

This review presents the current status of XR applications in cardiovascular care. The use of XR holds tremendous promise for transforming cardiology by increasing visualization, improving procedure planning, and revolutionizing medical education and rehabilitation. The number of publications is continuously increasing, and interesting approaches have been evaluated in almost every area of cardiology. Extended reality is offering several promising applications but also facing limitations. With the advancement of these technologies, cardiac healthcare outcomes may improve and the way cardiovascular diseases are diagnosed, treated, and managed could transform. Overcoming the obstacles mentioned above requires continuous research and solid validation through clinical studies. Only then can the full potential of XR in cardiology be unfolded.

## Supplementary Material

ztaf070_Supplementary_Data

## Data Availability

The anonymized data can be requested from the authors if required.
