# Supplementary material for: Extended reality in cardiovascular care: a systematic review
Source: Eur Heart J Digit Health. 2025 Jun 19;6(5):878–87. doi: 10.1093/ehjdh/ztaf070 (PMC12450517; doi:10.1093/ehjdh/ztaf070)
Supplement: ztaf070_Supplementary_Data [file ztaf070_supplementary_data.docx]

**Tables**

Table S1: Systematic review of extended reality in acute cardiovascular care

| **Authors (year, country)** | **Sample size (Intervention /control)** | | **Study type** | **Quality of evidence** | **VR user** | **Age (Mean ± SD) / Median (Range)** | **VR dosage (Frequency)** | **Timing of applying VR** | **Experimental group** | **Control group** | **Results** |
| --- | --- | --- | --- | --- | --- | --- | --- | --- | --- | --- | --- |
| **Valvular heart disease (N=23)** | | | | | | | | | | | |
| Bruno et al. (2020, Germany) (1) | 32 (16/16) | | RCT | 2 | Patients | 83 (78.25 – 87) | 30:30 minutes | Intraprocedural | Relaxing VR videos during TAVI | Standard care | The VR intervention group reported significantly less anxiety after the procedure (VAS 2 [IQR 0-3.75] vs 5 [IQR 2-8], p=0.04) than patients randomized to control. In the intervention group, 93.8% would use VR during TAVI again. |
| Lind et al. (2023, Germany) (2) | 117 (59/58) | | RCT | 2 | Patients | n/a | n/a | Intraprocedural | Relaxing VR videos during TAVI | Standard care | Post-interventional anxiety scores (STAI-S) (31.5 ± 13.4 vs. 38.5 ± 19.2, p = 0.02) and the perceived duration of the procedure (60.1 ± 32.3 vs. 73.0 ± 32.4, p = 0.04) were lower in the VR group. Procedure time, pain, and anxiety scores (visual analog scale) were similar between the groups. The complication rate was low and not associated with VR. Post-interventional delirium occurred in nine patients and was similar between the groups (VR: 4 [6.8%] vs. CG: 5 [8.6%], p = 0.71). No periprocedural strokes were observed. |
| Vanhoorebeeck et al. (2021, Belgium) (3) | 89 (30/39/12) | | RCT | 2 | Patient | 83.9 / 80.6 / 85.6 | n/a | Intraprocedural | Virtual reality  hypnosis (VRH) during TAVI | General anaesthesia or awake | Primary outcome parameters showed no differences except for reduced procedure time in awake and VRH groups. |
| Ruyra et al. (2022, Spain) (4) | 11(11/11) | | Observational study | 3 | Medical staff | n/a | n/a | Preprocedural planning | Planning of TAVI using VR | CT | VR models  modified the implant strategy in 5 cases (45.4% of the cases). |
| Carnahan et al. (Canada, 2020) (5) | 5 (5/5) | | Observational study | 3 | Medical staff | n/a | n/a | Preprocedural planning | VR system for the application of measuring chordal lengths from compounded TEE and TTE images | Traditional software | VR platform offered a more intuitive experience with respect to orientation, however users felt there was a lack of precision when performing the measurement tasks. |
| Narang et al. (2020, United states) (6) | 15 (15/15/15) | | Observational study | 3 | Medical staff | n/a | n/a | Anatomic measurements of mitral valve | VR visualization of mitral valve | 3D echocardiography (3DE) and CT | The measurement variabilities were reduced by 40% (20.1% vs 12.2%) for 3DE imaging and 34% (15.3% vs 10.1%) for CT imaging by using VR. The mean time needed for measurements was reduced by 31% (from 61 to 42 sec) for 3DE imaging and 39% (from 37 to 23 sec) for CT imaging. Most users reported facile manipulation of VR models, diagnostic quality visualization of the anatomy, and high confidence in the measurements. |
| Kanschik et al. (2024, Germany) (7) | 60 (60/60) | | Observational study | 3 | Medical staff | n/a | n/a | Preprocedural planning | VR for the planning of TAVI | CT | VR enables precise and reproducible measurements of the aortic valve and surrounding structures. In addition, VR visualization improves anatomical understanding and orientation. |
| Jolley et al. (2019, United States) (8) | 5 (5/5) | | Observational study | 3 | Medical staff | n/a | n/a | Preprocedural planning | VR modeling of catheter-based pulmonary valve replacement into native right ventricular outflow tracts | CT | VR modeling which assumes a rigid vessel  wall may not accurately predict the degree of distal RVOT expansion following actual device  placement. |
| Van den Bosch et al. (2005, Netherlands) (9) | 6 (6/0) | | Observational study | 3 | Medical staff | n/a | 10 min | Training | VR visualization of echocardiography data sets | n/a | Dynamic holographic imaging of three-dimensional  echocardiographic data is feasible. 10 independent observers correctly assessed the normal and pathological mitral valve in the holograms |
| Kamiya et al. (2022, Japan)  (10) | 1 (1/1) | | Observational study | 3 | Medical staff | n/a | n/a | Validation of the accuracy of virtual imaging | Aortic root and coronary artery cast measurement in VR | 2D-MPR | 3D-VR measurements seem considerably more accurate than the current standard 2D-MPR. 3D-VR may be considered the next gold standard for 3D measurement of cardiac anatomy in vivo |
| Kamiya et al. (2021, Japan) (11) | 1 (1/1) | | Observational study | 3 | Medical staff | n/a | n/a | Preprocedural | VR imaging analysis of the dynamic aortic root anatomy | Measurements of a known prosthesis | Measurement accuracy examined against a known prosthesis showed within 1 mm of error (less than 0.5%). |
| Kamiya et al. (2022, Japan) (12) | 1 (1/1) | | Observational study | 3 | Medical staff | n/a | n/a | Preprocedural | VR imaging of a dynamic, functioning aortic valve using an ex vivo porcine heart | Visual images seen through a fiberoptic scope | Superimposition of the images from the 2 different modalities showed the virtual reality images precisely matching the visual images in both systole and diastole |
| Currie et al. (2016, United States) (13) | 10 (10/10) | | Observational study | 3 | Medical staff | n/a | n/a | Preprocedural | AR guidance for TAVI | Fluoroscopic guidance | The precision of TAVI deployment using fluoroscopic guidance was 3.4 mm, whereas the precision of AR guidance was 2.9 mm, and its overall accuracy was 3.4 mm. |
| Chahine et al. (2024, United States) (14) | 22 (22/0) | | Observational study | 3 | Medical staff | n/a | n/a | Preprocedural planning | VR to predict paravalvular leak in bicuspid severe aortic valve stenosis in TAVI | n/a | VR model accurately predicted the presence and absence of PVL in all patients. The receiver operator characteristic curve showed an area under the curve of 0.83 (0.59-1.00, P = .03) for malposition in the VR-TAVI simulated model. |
| Zablah et al. (2024, United States) (15) | 27 (27/27) | | Observational study | 3 | Medical staff | n/a | n/a | Preprocedural planning | VR for screening for self-expanding  percutaneous pulmonary valves | Companies' dedicated analysis | The use of a self-expandable valve was recommended by companies' dedicated analysis in 23 cases (85.2%), by VR assessment in 26 cases (96.3), and finally implanted in 25 cases (92.6%). VR presented a good capacity to accurately classify nonsuitable patients (50% versus 100%). |
| Castellanos et al. (2020, United States) (16) | 46 (46/46) | | Observational study | 3 | Patients | >40 | 14.76 ± 6.34 | Education | Preprocedural 360°VR consultations for aortic valve stenosis (N = 37) and atrial fibrillation (N = 9) | Standard consultations | The satisfaction was significantly higher after the VR consultations. Patients also reported that experiencing 360° VR helped improve their understanding (98%) and comfort level (93%) with the proposed procedure. |
| Bonanni et al. (2024, Italy) (17) | 100 (100/100) | | Observational study | 3 | Medical staff | n/a | n/a | Preprocedural planning | Mixed Reality-based software for the planning of TAVI | CT | A higher level of agreement between the two software systems was observed for linear metrics The agreement was lower for area, perimeter, and annulus-to-coronary ostia distance measurements. Excellent interobserver reliability was demonstrated for most measurements, especially for direct linear measurements. |
| d'Aiello et al. (2024, Italy) (18) | 17 (17/17) | | Case series | 4 | Medical staff | n/a | n/a | Preprocedural planning | Holography-guided procedural planning for modifying Venus P-valve implantation | CT | The use of holographic models facilitated procedural planning in challenging anatomical scenarios. |
| Van De Bruaene et al. (2022, Belgium) (19) | 1 (1/1) | | Case report | 4 | Medical staff | n/a | n/a | Preprocedural planning | VR for planning of transcatheter mitral valve‐in‐valve replacement | CT | VR may provide  additional information since it provides malleable visualization of internal and external anatomy. |
| Sacha et al. (2022, Poland) (20) | 1 (1/1) | | Case report | 4 | Medical staff | n/a | n/a | Intraprocedural | AR for MitraClip implantation | TEE | The use of holography for MitraClip is feasible |
| Kasprzak et al. (2019, Poland) (21) | 1 (1/0) | | Case report | 4 | Medical staff | n/a | n/a | Intraprocedural | AR for percutaneous mitral balloon commissurotomy | n/a | Real-time intraprocedural use of holographic XR is feasible |
| Castellanos et al. (2022, United States) (22) | 1 (1/1) | | Case report | 4 | Medical staff | n/a | n/a | Preprocedural planning | VR for planning  percutaneous transseptal valve-in-valve  transcatheter mitral valve replacement | CT | Preoperative planning in VR alleviated concerns regarding obstruction of the neo-LVOT and helped confirm safe implantation by clearly showing the three-dimensional spatial relationship between the implants and surrounding patient anatomy |
| Sadri et al. (2024, United States) (23) | 24 (12/12) | | Proof-of-concept study | n/a | Medical staff | n/a | n/a | Intraprocedural | AR guidance for positioning of cerebral embolic protection devices during TAVI | Fluoroscopic guidance | AR guidance eliminated the need for aortic arch angiograms before device placement thus reducing contrast volume (0 mL vs 15 mL, P < 0.0001). There was no significant difference in the time required for filter placement or fluoroscopy time. Furthermore, AR guidance increased confidence in the wiring of the aortic arch and facilitated easier device placement. |
| **Non-valvular structural heart disease (N=12)** | | | | | | | | | | | |
| Pool et al.  (2022, Netherlands) (24) | 50 (25/25) | | RCT | 2 | Patients | 44.5 ± 9.9 /  43.1 ±12.0 | n/a | Education | VR video explaining PFO/ASD closure | Standard care | VR is effective in reducing pre-procedural anxiety in patients undergoing percutaneous PFO or ASD closure. |
| Heidari et al. (2023, Germany) (25) | 21 (21/21/21) | | Observational study | 3 | Medical staff | n/a | n/a | Preprocedural planning | VR for the planning of LAAC | CT, TEE | VR visualization of the LAA is feasible and allows precise and reproducible measurements in  planning of LAA closure procedures with enhanced 3D orientation |
| Pasquali et al. (2022, Italy) (26) | 4 (4/0) | | Observational study | 3 | Medical staff | n/a | n/a | Preprocedural planning | AR for the planning of LAAC | n/a | The morphological analysis of the holographic anatomical models was successfully applied for all the patients (n ​= ​4) independently from the morphology and it was performed in less than 10 minutes. |
| Mill et al. (2022, Spain) (27) | 5 (5/5) | | Observational study | 3 | Medical staff | n/a | 10 min | Preprocedural planning | VR for planning LAAC | Web-based 3D imaging visualization, computational fluid simulations, 3D printing | The results demonstrated the potential impact of advanced visual computing solutions and 3D printing to improve the planning of LAAC as well as the need for their integration into a single workflow to be used in a clinical environment. |
| Tejman-Yarden et al.  (2023, Israel)  (28) | 20 (20/20/20) | | Observational study | 3 | Medical staff | n/a | n/a | Preprocedural planning | VR for planning LAAC | CT, TEE | VR visualization of the LAA ostium from different perspectives allows for a better understanding of its funnel-shaped structure. VR measurement of the maximal ostium diameter had the strongest correlation with the diameter of the inserted device. |
| Heidari et al. (2024, Germany) (29) | 10 (10/10/10/10) | | Observational study | 3 | Medical staff | n/a | n/a | Preprocedural planning | VR for planning LAAC | 3D printing, CT, TEE | Device sizing was rated highest in MSCT (MSCT: 1.9 ± 0.8; TEE: 2.6 ± 0.9; 3D printing: 2.5 ± 1.0; VR: 2.5 ± 1.1; p < 0.01); TEE, VR, and 3D printing were superior in the visualization of the Fossa ovalis compared to MSCT (MSCT: 3.3 ± 1.4; TEE: 2.2 ± 1.3; 3D printing: 2.2 ± 1.4; VR: 1.9 ± 1.3; all p < 0.01). VR and 3D printing were superior in depth perception (VR: 1.6 ± 0.5; 3D printing: 1.8 ± 0.4; TEE: 2.9 ± 0.7; MSCT: 2.6 ± 0.8; p < 0.01).The visualization of extracardiac structures was rated less accurate in TEE than MSCT (TEE: 2.6 ± 0.9; MSCT: 1.9 ± 0.8, p < 0.01). |
| Sadeghi et al. (2021, Netherlands) (30) | 6 (6/6/6/6) | | Case series | 4 | Medical staff | n/a | n/a | Preprocedural planning | VR for planning transcatheter PVL closure | CT,TEE, and 3D computational models (3DCM) | VR measurements showed comparable results with CT/3DCMs. VR facilitated evaluation with in-depth perception and free 3D manipulation in space. |
| Vanreusel et al. (2020, Belgium) (31) | 1 (1/0) | | Case report | 4 | Patient | 66 | n/a | Intraprocedural | VR hypnosis during percutaneous closure of a secundum ASD | n/a | Positive experience for the patient |
| Shimura et al. (2023, Japan) (32) | 1 (1/0) | | Case report | 4 | Medical staff | n/a | n/a | Preprocedural planning | VR simulator for LAAC | n/a | Preprocedural VR simulation may be useful for procedural planning for patients with challenging anatomy undergoing LAAC |
| Zbronski et al. (2018, Poland) (33) | 1 (1/0) | | Case report | 4 | Medical staff | n/a | n/a | Pre- and Intraprocedural | AR for LAAC | n/a | AR use for LAAC is feasible |
| Zlahoda-Huzior et al. (2023, Poland) (34) | 1(1/1) | | Case report | 4 | Medical staff | n/a | n/a | Preprocedural planning | VR visualization of a complex ASD | CT | The virtual heart team was able to discern that the previously suspected multiple defect was in fact one large defect sequestered by the arcades. This finding was not perceived during the standard CT data analysis on flat screens. |
| Sawada et al. (2024, Japan) (35) | 1 (1/0) | | Case report | 4 | Medical staff | n/a | n/a | Preprocedural planning | VR simulator to visualize the ideal delivery sheath curve for LAAC | CT, TEE | With the help of the VR simulator, the original DS was reshaped to create the ideal curve, and the LAAC was successfully performed. |
| **Congenital heart disease (N=28)** | | | | | | | | | | | |
| Patel et al. (2021, United states) (36) | | 51 (24/27) | RCT | 2 | Medical  students, residents, fellows, nurses, advanced practitioners,  junior attending physicians, dieticians, and bioengineering PhD students | 18 - 64 | 34:20 min | Education | VR lecture about atrioventricular  canal | Desktop lecture | No statistically significant difference in the knowledge  acquisition; the VR group reported a better learning experience and self-assessment suggesting VR may increase learner  engagement in understanding CHD. |
| Wang et al. (2022, China) (37) | | 55 (28/27) | RCT | 2 | Patients | 24.5±2.55 / 24.7±1.83 | n/a | Education | VR-based preoperative notification to reduce anxiety in parents of children with simple CHD | Standard care | The state anxiety scale scores of participants in the VR group were lower than in the control group, and the difference was statistically significant (p < 0.05) |
| Kieu et al. (2023, United States) (38) | | 22 (22/0) | Observational study | 3 | Patients | 16 – 19 | 15 – 20 min | Transition visit | VR lessons about pulmonary stenosis, Tetralogy of Fallot, atrial and ventricular septal defect, coarctation, aortic stenosis, hypoplastic left heart syndrome, and patent ductus arteriosus | n/a | VR helped with understanding the heart lesion, and the heart surgery; the participants enjoyed the VR simulation and this was time well spent for them |
| Bertelli et al. (2023, Italy) (39) | 5 (5/0) | | Observational study | 3 | Five volunteers with no previous experience in the field of 3D reconstruction | n/a | n/a | Education | DIVA software was developed to simplify the VR modeling of five patients with right partial anomalous pulmonary venous return | n/a | All our participants recreated 3D models in a relatively short time, maintaining a good overall quality (average quality score ≥ 3 on a scale of 1-5). The overall trend of all the parameters analyzed showed a statistical improvement between case 1 and case 5, as users became more and more experienced. |
| Awori et al. (2023, United States) (40) | 30 (15/15) | | Observational study | 3 | Medical staff | n/a | 15 min | Education | VR models of a developmentally typical heart and tetralogy of Fallot pathology | 3D printing | Most participants had minimal prior exposure to VR (1.1 ± 0.4) or 3D printed models (2.1 ± 1.5). Participants endorsed a greater degree of understanding with VR models (8.5 ± 1) compared with 3D printed models (6.3 ± 1.8) or traditional models of instruction (5.5 ± 1.5) p < 0.001. Most participants felt comfortable with modern technology (7.6 ± 2.1). 87% of participants preferred VR over 3D printing. |
| Vegulla et al. (2023, United States) (41) | 3 (3/0) | | Observational study | 3 | Medical staff | n/a | n/a | Education | VR visualization of varied anatomic lesions | n/a | The ability to "walk through" different chambers of the heart makes the understanding of anatomy easy and intuitive. |
| Pajaziti et al. (2021, England) (42) | 58 (58/0) | | Observational study | 3 | Medical staff | n/a | 70 min | Education | VR models of 5 CHDs | n/a | VR has proven to be an effective tool for learning about congenital heart disease. In addition, there were use cases where VR offered novel learning experiences that were not possible with conventional methods. |
| Sun et al. (2005, China) (43) | 10 (10/0) | | Observational study | 3 | Medical staff | n/a | n/a | Preprocedural planning | Evaluation of 3D TTE  in congenital heart disease  such as ASD by VR | n/a | The site, geometry were well appraised in its true form. There were good correlations between the VR measurements and the independently measured anatomical data. |
| Topuzov et al. (2022) (44) | 20 (20/0) | | Observational study | 3 | Medical staff | n/a | n/a | Preprocedural planning | Assessment of aortic coarctation and stent simulation in VR | n/a | In 5 cases identical or very similar stents were proposed, in 12 cases simulations had slight, potentially avoidable misestimations either in stent length or diameter, and in 3 cases differences were more considerable. Overall, in 14 cases the location of the stent was concordant between the simulation and reality, and in the remaining 6 cases the simulated stent was located lower than the actual one. |
| Lim et al. (2023, United States) (45) | 132 (52/80) | | Observational study | 3 | Medical staff | n/a | n/a | Training | VR modules for six common congenital heart lesions | Standard training | Trainees in the VR group achieved higher scores on the assessment (20.4 ± 2.9 versus 18.8 ± 3.8 out of 27 questions answered correctly, p = 0.01). Further analysis showed significant improvement in the VR group for questions specifically testing visuospatial concepts. All users recommended the integration of the program into the residency curriculum. |
| Kang et al. (2020, Canada) (46) | 3 (3/3) | | Observational study | 3 | Medical staff | n/a | n/a | Education | MR models of different subtypes of double-outlet right ventricle | Macroscopic evaluation | The results showed that the MR system provided highly accurate stereoscopic display of spatially complex congenital cardiac lesions, with interactive features that might enhance 3-D understanding of morphology. |
| Ghosh et al.  (2022, United States) (47) | 112 (112/0) | | Observational study | 3 | Medical staff | n/a | n/a | Education and preprocedural planning | VR visualization of anatomy | n/a | Clinical application of 3D modeling can be seamlessly integrated into preprocedural care for patients with CHD. The most common indications for modeling were complex biventricular repair (n = 30, 31%) and repair of multiple VSD (n = 11, 12%). |
| D'Aiello et al. (2023, Italy) (48) | 59 (20/20/19) | | Observational study | 3 | Medical students | n/a | 30 min | Education | Interaction directly with holographic  anatomical models | traditional slides or slides incorporating videos of holographic anatomical models | Holographic images helped students in better understanding the technical aspects and possible complications of SV-ASD transcatheter treatment. |
| Xue et al. (2010, China) (49) | 40 (40/40) | | Observational study | 3 | Medical staff | n/a | n/a | Education | VR visualization of ASD, VSD, TOF, and DORV | 2D echocardiographic (2DE) | Compared with The diagnostic accuracy of VE was significantly higher than that of 2DE for TOF and DORV except for ASD and VSD. The receiver operating characteristic (ROC) curve for VE was closer to the optimal performance point than was the ROC curve for 2DE. 3D echocardiographic VE can enhance our understanding of intracardiac structures and facilitate the evaluation of congenital heart disease. |
| Lau et al. (2021, Australia) (50) | 29 (29/29) | | Observational study | 3 | Medical staff | n/a | 15 min | Education | VR visualization of four selected CHD cases | 3D printing | VR was perceived as more useful in medical education and preoperative planning compared to 3D printed heart models, although there was no significant difference in the ratings (p = 0.54 and 0.35, respectively). Twenty-one participants (72%) indicated both the VR and 3DPHM provided additional benefits compared to the conventional medical imaging visualizations. |
| Kim et al. (2021, United states) (51) | 22 (22/22) | | Observational study | 3 | Medical trainees | n/a | n/a | Education | VR visualization of ASD, CoA, and MAPCA | Conventional 2D display | Diagnostic accuracies were highest when groups used the full-immersive VR compared with the conventional and  nonimmersive VR. The full-immersive VR was ranked as the most preferred. |
| Lau et al. (2022, Australia) (52) | 34 (34/34) | | Observational study | 3 | Medical staff | n/a | n/a | Education | MR models of complex CHD | 3D-printed heart models and DICOM images | MR models were ranked as the best modality in demonstrating complex CHD lesions (mean difference (MD) = 0.76, p = 0.01), in enhancing depth perception (MD = 1.09, p = 0.00), in portraying spatial relationship between cardiac structures (MD = 1.15, p = 0.00), as a learning tool of the pathology (MD = 0.91, p = 0.00), and in facilitating pre-operative planning (MD = 0.87, p = 0.02). The 3DPHM were the best modality in facilitating communication with patients (MD = 0.99, p = 0.00). |
| Giffoni et al. (2024, Brazil) (53) | 12 (12/0) | | Observational study | 3 | Medical staff | n/a | n/a | Preprocedural planning | VR platform for fetal heart segmentation | n/a | VR offered improved visuospatial visualization and detailed anatomical insights |
| Salavitabar et al. (2024, United States) (54) | 2 (2/2) | | Case series | 4 | Medical staff | n/a | n/a | Preprocedural planning | VR for planning of  complex percutaneous congenital interventions | CT | VR provides an advanced approach to 3D visualization of CTA while providing a virtual environment for remote collaboration. Using CT-generated devices, it is possible to simulate virtual device implantations. |
| Mejia et al. (2024m United States) (55) | 2 (2/2) | | Case series | 4 | Medical staff | n/a | n/a | Procedural planning | VR for planning ductus venosus stenting in obstructed total anomalous pulmonary venous return | CT | VR may be beneficial in providing valuable insight to the ductus venosus anatomy and allow clinicians further insight and confidence for successful procedural planning and guidance. |
| Tandon et al. (2019, United states) (56) | (1/1) | | Case report | 4 | Medical staff | n/a | n/a | Procedural planning | VR for planning transcatheter closure of sinus venosus defect | CT, CMR | VR environment provided 3D information about anatomic relationships that were  not immediately obvious on 2D imaging and thus provides an excellent platform for planning  patient-specific interventions in CHD. |
| Kasprzak et al. (2019, Poland) (57) | 1 (1/0) | | Case report | 4 | Medical staff | n/a | n/a | Intraprocedural | XR for percutaneous patent ductus arteriosus closure | n/a | The use of intraprocedural XR holographic display is feasible |
| Ghosh et al. (2021, United States) (58) | 1 (1/0) | | Case report | 4 | Medical staff | n/a | n/a | Preprocedural planning | Visualization of Multiple Ventricular Septal Defects | n/a | Viewing the models in VR allowed the team to precisely locate the defects and decide on a hybrid transcatheter and surgical approach  to ensure a successful repair. |
| Sivakumar et al. (2024, India) (59) | 1 (1/1) | | Case report | 4 | Medical staff | n/a | n/a | Preprocedural planning | VR for planning transcatheter closure of sinus venosus defects | CT | VR enables precise and fast simulations of stent implantation |
| Szeliga et al. (2024, Poland) (60) | 1 (1/1) | | Case report | 4 | Medical staff | n/a | n/a | Preprocedural planning | VR for planning an intervention in a child with aneurysms after numerous interventions for the treatment of a hypoplastic aortic arch and coarctation. | CT | VR enabled detailed imaging of the aortic arch and its surroundings, what facilitated the planning. |
| Wilson et al. (2024, United States) (61) | 1 (1/1) | | Case report | 4 | Medical staff | n/a | n/a | Preprocedural planning | VR for planning transcatheter pulmonary valve replacement (TPVR) | CT | Particularly in borderline cases, VR may contribute to a more comprehensive interpretation of the patient's anatomy when determining candidacy for TPVR. |
| Dziewulska et al. (2024, Poland) (62) | 1 (1/1) | | Case report | 4 | Medical staff | n/a | n/a | Preprocedural planning | VR for planning of percutaneous first step palliation in a newborn with heterotaxy syndrome | CT | The interventionalists were of the opinion that planning would not be so easy without the use of VR |
| Galeczka et al. (2023, Poland) (63) | 1 (1/1/1) | | Case report | 4 | Medical staff | n/a | n/a | Procedural planning | Visualization of the left pulmonary artery stenosis | CT, MRI | VR enabled visualization  of the LPA stenosis from the outside and the  inside of the pulmonary artery.  Moreover, a simulation of LPA stenting was performed,  and angiography projections were  planned |
| **Cardiac catheterization (N=35)** | | | | | | | | | | | |
| Larsson et al. (2023, France) (64) | 139 (62/77) | | RCT | 2 | Patients | 62.5 (10.9) /  62.6 (9.5) | 20 min | Preprocedural | Use of a VR mask in the transfer room before invasive coronary angiography | Standard care | No statistical difference in SDNN was observed between the VR and control groups (45.5 ± 17.8 vs 50.6 ± 19.5, p = 0.12). |
| Gökce et al. (2023, (65) | 153 (51/51/51) | | RCT | 2 | Patients | 59.4 ± 12.1 / 58.2 ± 13.4 /  58.2 ± 12.0 | 30 min | Intraprocedural | Use of VR during coronary angiography. | Acupressure or control group | Both intervention groups had significantly lower pain and anxiety scores, as well as higher comfort scores, compared to the control group (p < 0.001). The VR group had lower systolic blood pressure, respiratory rate and pulse rate than the control group (p < 0.05). The acupressure group had lower systolic and diastolic blood pressure and respiratory rate than the control group (p < 0.05). |
| Morgan et al. (2021, United Kingdom) (66) | 64 (33/31) | | RCT | 2 | Patients | 68.7 (38-84) | 10 min | Preprocedural | VR video describing the preprocedural and procedural experience for the day of their cardiac catheterization | Standard care | The VR group had a significantly greater reduction in anxiety level from baseline to post procedure than the control group (-5.1 vs -4.0, respectively; P=.03). In addition, the VR group had a better procedural understanding (3.88 vs 3.23, respectively; P<.01) and higher overall satisfaction than the control group (9.35 vs 8.97, respectively; P=.04). |
| Popovic et al. (2019, France) (67) | 20 (10/10) | | RCT | 2 | Medical staff | n/a | n/a | Training | VR simulation training in coronary angiography | Standard training | In real-life cases, the procedure time was shorter (p = 0.002), the radiation dose lower (p = 0.001), and the global procedure skill score was higher (p = 0.0001) in the VR group as compared with the control group. During virtual training procedural time (p <0.001), fluoroscopic time (p <0.001), training contrast amount (p <0.001), and global training score (p <0.001) significantly decreased. In the control group, all monitoring procedure parameters were significantly improved after trainig, as well as, the global procedure flow score (p <0.0001). |
| Cates et al. (2016, United states) (68) | 12 (6/6) | | RCT | 2 | Medical staff | 48 (34 – 68) | n/a | Training | VR stimulator for learning carotid artery angiography (CA) | Standard training | Experienced interventional cardiologists trained  on the VR simulator performed significantly better than their equally experienced controls showing a significantly lower rate of objectively assessed intraoperative errors in CA. |
| Voelker et al. (2016, Germany) (69) | 18 (9/9) | | RCT | 2 | Medical staff | n/a | 7:30 h | Training | VR simulator to practice coronary interventional procedures | Standard training | The "skills score" increased by 5.8 ± 6.1 points in the VR simulation group and decreased by 6.7 ± 8.4 in the control group (P = 0.003) from the simple stenosis at pre- to the more complex lesion at post-evaluation demonstrating the effectiveness of simulation-based training. |
| Jensen et al.  (2016, Sweden) (70) | 16 (8/8) | | RCT | 2 | Medical staff | 36 | 10 h | Training | VR training in a coronary angiography | Standard training | VR-trained group had shorter fluoroscopy and total procedure times than the controls (median 558 vs. 842 seconds, p=0.003 and 1,356 vs. 1,623 seconds, p=0.032, respectively). The controls had a higher error score (median 27 vs. 15, p=0.002) and a lower performance score (median 47 vs. 68, p=0.006) than the VR-trained residents. |
| Bagai et al. (2012, Canada) (71) | 27 (12/15) | | RCT | 2 | Medical staff | 29 (28.5 - 32) | 2 – 4 hours | Training | VR simulator for cardiac catheterization | Standard training | Technical performance improved postintervention in the simulator group (24 versus 18; P=0.008) and changed marginally in the control group (20 versus 18; P=0.054). Improvement in technical performance was greater in the VR group (6 versus 1; P=0.04). Global performance improved postintervention in both groups (simulator, 24 versus 17, P=0.01; control, 20 versus 18, P=0.02), with a trend toward greater improvement in the simulator group (5 versus 2; P=0.11). |
| Aeckersberg et al. (2019, Germany) (72) | 50 (26/12/12) | | RCT | 2 | Medical students | n/a | n/a | Training | VR simulators in basic endovascular skills training | Conventional learning through a video podcast or low fidelity simulation training with tablet  paired touch-gesture navigation | Whereas VR simulator training was associated with increased confidence of trainees in their skills, assessment of their practical skills showed no actual improvement. |
| Perez-Gutierrez et al. (2020, Colombia) (73) | 10 (5/5) | | RCT | 2 | Medical students | n/a | n/a | Training | VR percutaneous  coronary intervention simulation for acute myocardial infarction | Non-immersive simulation | The visual immersion and non immersion effects are compared by gathering usability perceptions finding that the simulator has potential as a learning tool for medical education using VR technology. |
| Willaert et al. (2012, Belgium) (74) | 20 (20/20/20) | | Randomized crossover | 3 | Medical residents | 28 (25–37) | n/a | Training | VR generic carotid artery stenting (CAS) warm-up | Patient-specific simulated rehearsal (PsR) of a carotid artery stenting procedure | Performances were significantly better after PsR than after a generic warm-up or no warm-up for total procedure time (16.3 ± 0.6 vs 19.7 ± 1.0 vs 20.9 ± 1.1 minutes, P = 0.001) and fluoroscopy time (9.3 ± 0.1 vs 11.2 ± 0.6 vs 11.2 ± 0.5 minutes, P = 0.022) but did not influence contrast volume or number of roadmaps used during the "real" case. PsR significantly improved the quality of performance as measured by the expert-based ratings (scores 28 vs 25 vs 25, P = 0.020). |
| Van Herzeele et al. (2007, London) (75) | 45 (45/0) | | Observational study | 3 | Medical staff | n/a | n/a | Training | VR simulator for carotid artery stent procedures | n/a | Total time and fluoroscopic time both recorded by a realistic VR simulator differentiate between levels of CAS experience in experienced interventionalists. |
| Li et al. (2021, China) (76) | 36 (36/36) | | Observational study | 3 | Medical staff | n/a | n/a | Training | VR based simulation system for PCI | Traditional desktop intervention simulators | VR simulation system can provide a better user experience, and is a suitable platform for PCI surgery training and rehearsal |
| Jensen et al. (2014, Sweden) (77) | 100 (54/46) | | Observational study | 3 | Medical staff | n/a | n/a | Training | VR based course in coronary angiography | Standard training | No consistent acceleration in the early learning curve. VR group had longer fluoroscopy time (median 360 seconds (IQR 245-557) vs. 289 seconds (IQR 179-468), p < 0.001). Safety measures also indicated more complications appearing at the ward, in particular when using the femoral approach (6.25% vs. 2.53%, p < 0.001). |
| Aardoom et al. (2022, Netherlands) (78) | 8 (8/0) | | Observational study | 3 | Patients | 67 ± 7.5 | 20 min | Patient education, preprocedural | 360° videos and interactive photos for patients undergoing cardiac catheterization. | n/a | Participants reported high levels of presence in the virtual environment The usability of Pre-View was well evaluated and patient satisfaction was high. Usability and satisfaction scores were higher for participants who underwent Pre-View at home versus those who underwent Pre-View at the hospital, although the latter group was significantly older; 72.8 versus 61.3, respectively. All participants reported Pre-View to be effective in terms of feeling better informed about the care process of cardiac catheterization. Most participants (7/8, 88%) reported Pre-View to be effective in terms of feeling better prepared for cardiac catheterization, acknowledging the potential of Pre-View in reducing negative psychological consequences after catheterization. |
| Nicholson et al. (2006, United states) (79) | 100 (100/0) | | Observational study | 3 | Medical staff | n/a | 4 h | Training | VR simulation for carotid angiography | n/a | Anatomically, physicians reported that the aortic arch and carotid vasculature were well simulated (mean = 4.4). However, the bony structures and intra-cranial vasculature correlated poorly with actual anatomic structures (means = 2.3 and 1.1, respectively). The simulated sequence of using guidewires and catheters was rated to be nearly identical to actual CA (means = 4.8). |
| Vardhan et al. (2019, United States) (80) | 31 (31/31) | | Observational study | 3 | Medical staff | n/a | n/a | Preprocedural | VR based computational fluid dynamics with full immersive display | VR based computational fluid dynamics with semi immersive display | CFD-based VR system rendered on semi-immersive displays can enable more accurate and efficient stent placement. |
| Räder et al. (2014, Denmark) (81) | 10 (10/10) | | Observational study | 3 | Medical staff | n/a | n/a | Training | VR simulator for coronary angiography | Training in catheterization laboratory | The association between CA performance in a simulated setting and performance in the catheterization laboratory was not linear. Familiarity with the simulator may overestimate proficiency. |
| Popovic et al. (2022, France) (82) | 10 (10/0) | | Observational study | 3 | Medical staff | 26.1 ± 1.8 | 4 h | Training | VR simulator for coronary angiography | n/a | VR simulator for coronary angiography may improve radioprotection learning and should be incorporated into training curricula. |
| Patel et al. (2006, United states) (83) | 20 (20/0) | | Observational study | 3 | Medical staff | 62.9 ±  10.3 | n/a | Training | VR simulation training for carotid stenting | n/a | An improvement was noted in procedure time, contrast volume, fluoroscopy time, and composite catheter handling errors when comparing the subjects' first and last simulations (all p < 0.05). |
| Li et al. (2021, China) (84) | 36 (36/36) | | Observational study | 3 | Medical staff | n/a | n/a | Training | VR simulation system for personalized percutaneous coronary intervention | Traditional desktop intervention simulators | VR simulation system can provide a better user experience, and is a suitable platform for PCI surgery training and rehearsal |
| Salavitabar et al. (2023, United states) (85) | 30 (30/30) | | Observational study | 3 | Medical staff | n/a | n/a | Periprocedural | AR visualization of 3D Rotational Angiography in Congenital Heart Disease | Standard visualization | Visualization and identification of structures were graded as “very easy” in 81.1%  (n=73) and 67.8% (n=61) of AR and CM, respectively. Fifty-nine (66%) grades ‘Agreed’ or ‘Strongly Agreed’ that AR models provided superior appreciation of 3D relationships; AR was found to be least beneficial in visualization of aortic arch obstruction. AR models were thought to be helpful in identifying pathology and assisting in interventional planning in  85 assessments (94.4%). |
| Zhang et al. (2022, China) (86) | 2146 (2146/2146) | | Observational study | 3 | Medical staff | n/a | n/a | Postprocedural | Post-PCI outcomes predicted by pre-intervention simulation of residual quantitative flow ratio using AR | Post-PCI QFR | Simulated residual QFR and post-PCI QFR were strongly correlated (r = 0.976). Low simulated residual QFR (≤0.92) was independently associated with higher risk of 2-year vessel-oriented composite endpoint (adjusted hazard ratio: 5.50; 95% confidence interval: 3.03 to 10.0). |
| Opolski et al. (2017, Poland) (87) | 74 (15/59) | | Observational study | 3 | Medical staff | n/a | n/a | Intraprocedural | AR for percutaneous revascularization of CTO | CTO PCI without AR | CTA-assisted CTO PCI using an AR glass is feasible and safe, and might reduce the resources required for the interventional treatment of CTO. |
| Alonso-Felipe et al. (2023, Spain) (88) | 2 (2/0) | | Observational study | 33 | Medical staff | n/a | n/a | Intraprocedural | XR to ultrasound‑guided Femoral  Arterial Cannulation | n/a | Easy to use, reliable, realtime, reachable, and cost-efective, allowing a reduction of operating times, a better control of typical errors associated to the interventional procedure, and opening the possibility to use the medical imagery produced in ubiquitous e-learning |
| Bloom et al. (2022, United states) (89) | 30 (30/0) | | Observational study | 3 | Medical staff | n/a | n/a | Training | XR ultrasound system for vascular puncture | Conventional | XR resulted in an overall reduction in number of needle repositions and improvement in quality of access as measured by distance and angle of elevation. These findings were even more evident in the right femoral vein access site. Use of MantUS resulted in faster time to access, fewer number of both access attempts and number of needle repositions compared to conventional US. Postparticipant survey showed high levels of usability (87%) and a belief that MantUS may decrease adverse outcomes (73%) and failed access attempts (83%). |
| Chahine et al. (2022, United states) (90) | 50 (50/50) | | Observational study | 3 | Medical staff | n/a | n/a | Intraprocedural | XR head-mounted displays during right heart catheterizations and coronary angiographies | Standard treatment | The use of XR associated with a significantly lower procedure time (20 min (IQR 14–30) vs. 25 min (IQR 18–36), p = 0.038). There were no significant differences in median fluoroscopy time (1.5 min (IQR 0.7–4.9) in the study group vs. 1.3 min (IQR 0.8–3.1), p = 0.84) or median DAP (165.4 mGy·cm2 (IQR 13–15,583) in the study group vs. 913 mGy·cm2 (IQR 24–6291), p = 0.17). There was no significant increase in intra- or post-procedure complications. |
| Salavitabar et al. (2022, United states) (91) | 5 (5/0) | | Case series | 4 | Medical staff | n/a | n/a | Intraprocedural | AR visualization of 3D rotational angiography in  congenital cardiac catheterization | n/a | Intraprocedural AR visualization of 3D rotational angiography in patients with CHD is feasible |
| Witkowski et al. (2019, Poland) (92) | 2 (2/0) | | Case series | 4 | Medical staff | n/a | n/a | Intraprocedural | AR for percutaneous interventions on pulmonary arteries | n/a | AR use for pulmonary arteries interventions is feasible |
| Zablah et al. (2023, United states) (93) | 3 (3/3) | | Case series | 4 | Patients | 15 | n/a | Intraprocedural | VR system to distraction the patient during the intervention | Standard care | Virtual reality system helps to reduce the anxiety and discomfort |
| Higami et al.  (2023, Japan) (94) | 1 (1/0) | | Case report | 4 | Medical staff | n/a | 5-10 min | Preprocedural planning | VR-guided guiding catheter (GC) simulation before PCI | n/a | VR-guided GC simulation has the potential to solve the catheter approach difficulty for any cardiovascular intervention |
| Sadeghi et al. (2021, Netherlands) (95) | 1 (1/0) | | Case report | 4 | Medical staff | n/a | 10 min | Preprocedural planning | VR-based  remote multidisciplinary coronary revascularisation  heart team meetings | n/a | VR-based meetings were rated as an easy-to-use, useful and effective method for remote heart team meetings. |
| Roguin et al.  (2012, Israel) (96) | 1 (1/0) | | Case report | 4 | Medical staff | n/a | n/a | Training | VR visualization prior to carotid artery stenting | n/a | A case rehearsal prior to an intervention may be useful in the planning and execution of carotid artery stenting |
| Opolski et al. (2015, Poland) (97) | 1 (1/0) | | Case report | 4 | Medical staff | n/a | n/a | Intraprocedural | Visualization of coronary vessels | n/a | The VR projection allowed the  operators to clearly visualize the distal coronary vessel, and verify the direction of the guide wire advancement relative to the course of the occluded vessel segment |
| Goto et al. (2024, Japan) (98) | 1 (1/0) | | Case report | 4 | Medical staff | n/a | n/a | Preprocedural planning | VR-guided guiding catheter (GC) simulation before PCI | n/a | VR-guided GC simulation is a useful new option that can help visualize the anatomy and ensure safe procedures for complex lesions |
| **General training, patient education, and heart failure (N=19)** | | | | | | | | | | | |
| Groninger et al. (2021, United states) (99) | 88 (52/36) | | RCT | 2 | Patients | 56.1 ±  13.2 | 10 min | During the hospital stay | 10-minute VR guide through a forest and  waterfall with voice narration | 2D guided imagery active control | Significant improvement in pain score after either 10 minutes of VR (change from pre- to post -2.9 ± 2.6, p < 0.0001) or 10 minutes of guided imagery (change from pre- to post -1.3 ± 1.8, p = 0.0001); VR led to greater reduction in pain score compared to guided imagery (p = 0.0011). Total quality-of-life and general distress scores did not significantly change for either arm. 89% participants responded that they would be willing to use the assigned intervention again. |
| Herbert et al. (2021, United States) (100) | 33 (19/14) | | RCT | 2 | Nursing students | n/a | n/a | Education | AR app on heart failure | Video lecture | There were no significant differences in learning, although more students indicated that they preferred the AR app. |
| Jiravska Godula et al. (2023, Czech Republic) (101) | 182 (94/88) | | RCT | 2 | Patients | 66 | 8 min | Education | 3D  educational video about hypertension | Traditional physician-led education | Median objective scores were significantly higher for VR (14, IQR 3) compared to traditional education (10, IQR 5), p < 0.001, indicating superior hypertension knowledge acquisition with VR. Subjective satisfaction was high in both groups. |
| Hessabi et al. (2020, Iran) (102) | 60 (30/30) | | RCT | 2 | Patients | n/a | 15 min | Impact on anxiety level in patients hospitalized in cardiac care units | VR videos of natural landscapes | Standard care | The mean anxiety score of the intervention group was significantly decreased after the intervention (P <0.001). There was also a significant difference between the mean score of anxiety in the intervention group and the control group (P <0.001) |
| Li et al. (2024, China) (103) | 148 (70/78) | | RCT | 2 | Patients | 58.3 (9.3) / 59.9 (9.5) | 30 min every  day for a total of 7 days | Education | VR-based cognitive-behavioural therapy (VR-CBT) for patients with acute myocardial infarction | Standard mental health support | VR-CBT reduced the anxiety compared with standard mental health support alone in terms of HAM-A scores at both post intervention (Cohen's d=-1.27 (95% confidence interval (CI): -1.64 to -0.90, p<0.001) and 3-month follow-up (Cohen's d=-0.37 (95% CI: -0.72 to -0.01, p=0.024). 88.6% paticipants completed completed the entire intervention. Cybersickness was the main reported adverse event (n=5). |
| Behera et al. (2024, United States) (104) | 61 (31/30) | | RCT | 2 | Patients | 10.9±3.3 / 11.3±3.9 | During TTE examination, 46.6±16.1 | Intraprocedural | VR system playing a television show or movie | Standard examination | Anxiety (average CAM-S difference 0.78 ± 1.80, p = 0.0012) and fear (average CFS difference 0.36 ± 0.74, p = 0.0005) decreased in both groups. There was no difference between groups in the change in anxiety and fear (p = 0.96-1.00). TTE study comprehensiveness and diagnostic accuracy were high in both groups. Procedure time was less for the VR group (48.4 ± 18.1 min) than the non-VR group (58.8 ± 24.4 min), but without a statistically significant difference (p = 0.075). |
| Choi et al. (2024, Korea) (105) | 22 (11/11) | | RCT | 2 | Medical stuff | 30.5±3.6 / 32.1±4.0 | n/a | Training | AR glasses for visual estimation of ejection fraction in patients with heart failure | Estimation the visual ejection fraction of  echocardiographic video clips without the AR glasses | The ICC with glasses (0.969, 95% CI 0.966 to 0.971) was higher than without glasses (0.705, 95% CI 0.681 to 0.727) among all participants. The first-year and second-year residents showed the most significant difference, with an ICC of 0.568 (95% CI 0.508 to 0.621) without glasses compared with 0.963 (95% CI 0.958 to 0.968) with glasses. For the third-year and fourth-year residents group, the ICC was 0.754 (95% CI 0.720 to 0.784) without glasses and 0.972 (95% CI 0.958 to 0.968) with glasses. Among the group of attending physicians, the ICC was 0.807 (95% CI 0.775 to 0.834) without glasses and 0.973 (95% CI 0.969 to 0.977) with glasses. |
| Nas et al. (2020, Netherlands) (106) | 381 (190/191) | | RCT | 2 | Medical and no medical personal | 26 (22-31) | n/a | Training | VR CPR training | Face-to-face CPR training | VR training resulted in noninferior chest compression rate but inferior compression depth; CPR scenario performance and chest compression fraction were worse, but leaning was less common than in face-to-face training. |
| Bruining et al. (1999, Netherlands) (107) | 61 (61/0) | | Observational study | 3 | Medical staff | n/a | n/a | Training | VR heart model based on TTE and TEE data | n/a | A VR heart model was successfully implemented in 3-D reconstruction software and can be used as an orientation tool and for teaching purposes. |
| Nielsen et al. (2022, Denmark) (108) | 36 (36/0) | | Observational study | 3 | Medical staff | n/a | n/a | Training | VR simulation-based test for assessing  skills in echocardiography | n/a | The test had a high consistent reliability (Alpha = .81), 95% CI [.69, .89]. In both cases, the performers' level was consistent, fitting others at the same level of experience (intraclass correlation r (35)=.81, p<.001). A pass/fail score of 48/50 points was established based on the mean test score of novice and experienced physicians. |
| Forgiarini et al. (2024, Italy) (109) | 17 (17/0) | | Observational study | 3 | Medical staff | n/a | n/a | Training | VR system for STEMI training | n/a | The VR system was appreciated (n = 17) and described as engaging (n = 4), challenging (n = 5), useful to improve self-confidence (n = 4), innovative (n = 5), and promising for training courses (n = 10) |
| O’Sullivan et al. (2021, Ireland) (110) | 15 (15/15) | | Observational study | 3 | Medical staff | n/a | n/a | Training | VR echocardiography | Live echocardiography demonstration | VR echocardiography is a useful teaching tool with 87% (n=13) rating it as good or very good on a 5-point Likert scale. When asked to compare VR to live demonstration, 67% (n=10) rated VR echocardiography as the same or better than live demonstration. One of the participants reported a side effect - mild and self-resolving dizziness. VR echocardiography is a safe, inexpensive and practical way for trainees to learn echocardiography. |
| Balian et al. (2019, United states) (111) | 51 (51/0) | | Observational study | 3 | Medical staff | 31 (27–41) | n/a | Training | AR CPR training system | n/a | 82% perceiving the experience as realistic, 98% recognizing the visualizations as helpful for training, and 94% willing to use the application in future CPR training. |
| Mehrpooya et al. (2024, Iran) (112) | 60 (20/20/20) | | Observational study | 3 | Medical students | n/a | n/a | Education | Routine method plus micro-video delivery in an AR infographic poster | Routine method or routine method plus offline micro-video delivery | The highest post-test score was for the offline micro-video delivery group and pairwise comparisons of post-test scores showed a significant difference between this group and the control one (p-value = 0.013). Additionally, the AR infographic poster group had the highest satisfaction score (p-value = 0.000). |
| Hilt et al. (2021, Netherlands) (113) | 22 (12/10) | | Observational study | 3 | Patients | 60 ±  9.5) | n/a | Education | XR to illustrate the function of statin treatment after myocardial infarction | Standard care | Ten out of 12 (83%) patients in the intervention group improved their statin knowledge by using the MR application (median 8 points, IQR 8). Test improvement was mainly the result of increased understanding of statin mechanisms in the body and secondary preventive effects. A high tendency to get involved and focused in virtual environments was moderately positive correlated with better test improvement (r = 0.57, P < 0.05). The median post-test score in the control group was poor (median 6 points, IQR 4). |
| Hilt et al. (2020, Netherlands) (114) | 18 (18/0) | | Observational study | 3 | Patients and medical staff | 62.7 ± 10.4 | n/a | Education | XR for  information and education after MI | n/a | Improving medication knowledge seems to be a feasible target for XR. |
| Galvez et al. (2021, United States) (115) | 32 (32/0) | | Observational study | 3 | Students | n/a | n/a | Education | VR support for learning the peripheral and collateral circulation | n/a | VR proved to be an effective learning approach |
| Skalidis et al. (2024, Greece) (116) | 3 (3/0) | | Observational study | 3 | Patients | n/a | n/a | Training | Metaverse-based CMR simulation for claustrophobic patients | n/a | After the training all patients entered the machine without issues and with reduced anxiety. Patients found the training useful, suggesting platform familiarization. |
| Yogev et al. (2022, Israel) (117) | 20 (20/20) | | Proof-of-concept study | n/a | Medical staff | n/a | n/a | Training | Semiautomated VR modeling for volumetric analysis of the heart ventricles | Standard dedicated cardiac imaging platform (PDP) | Comparison of the VR and the PDP left ventricle volumes demonstrated excellent correlation (95% CI 0.96, 0.99), and a small mean difference and range. The calculated volumes of the right ventricle had a somewhat lower correlation of 0.89 (95% CI 0.781, 0.95), a small mean difference, and a broader range. The VR chamber size estimations were significantly smaller than the estimates based on the PDP. |
| **Electrophysiology (N=19)** | | | | | | | | | | | |
| De Ponti et al. (2011, Italy) (118) | 14 (7/7) | | RCT | 2 | Medical staff | 31 | 4 days (3-64) | Training | VR simulator for performance of transseptal catheterization | Conventional training | VR training with results in shorter training times and superior post-training performance. |
| Chang et al.  (2021, Taiwan) (119) | 33 (11/22) | | RCT | 2 | Patients | n/a | 3 min | Preprocedural education | Interactive VR educational material | Paper-based materials | VR improved patients’ self-assessed self-efficacy on AF ablation knowledge. At the posteducation stage, the patients’ satisfaction to preprocedural education and used materials were higher among the VR group.  VR materials increased the effectiveness of education and preparedness for AF catheter ablation. Operators subjectively reported that the periprocedural cooperation  was increased both among paper and VR group patients after preprocedural education. Better preparedness of VR group patients was supported by less periprocedure pain, anxiety, and impatience. |
| Gorbaty et al.  (2023, United State) (120) | 18 (18/18) | | Observational study | 3 | Medical staff | 35 ± 4.5 | 40 min | Training | Teaching cardiac anatomy with VR | Traditional lecture-based teaching cardiac anatomy | VR experience were rated positively and preferred to the traditional teaching method; one-third of participants rating the system as hard to use; No learning advantage demonstrated over traditional lecture format. |
| Hermans et al. (2023, The Netherlands) (121) | 134 (68/66) | | Observational study | 3 | Patients | 66 (58–72 | 4:18 min | Patient education | Standard information and a VR video about ablation procedure | Standard preprocedural information | The number of patients that worried about the ablation procedure was lower in VR than in control patients (19.1% vs. 40.9%, P = 0.006). More VR females than males had worries about the procedure (34.8% vs. 11.1%, P = 0.026). The number of VR patients that were satisfied with the preprocedural information provision was higher post-ablation than pre-ablation (83.3% vs. 60.4%, P = 0.007). In total, 59.4% reported that the disposable cardboard was easy to use and led to a discussion with relatives in 68.8%. |
| Coulibaly et al. (2022, France) (122) | 86 (25/61) | | Observational study | 3 | Patients | 66 ±16 | n/a | Intraprocedural | Short video and audio sequences with a hypnotic and relaxing impact during electrophysiology and pacing procedures under conscious sedation | Standard treatment | There were no differences in procedure duration (46 (±29) vs. 56 (±32) min, p = 0.18) or in hypnotic/antalgic consumption (midazolam 1.95 (±1.44) vs. 2.00 (±1.22) mg, p = 0.83; sufentanyl 3.78 (±2.87) vs. 3.58 (±2.48) μg, p = 0.9) between the control and VR groups. In a multivariate analysis, the use of VR was independently associated with lower comfort during the procedure assessed by postoperative visual analogue scale (OR 15.00 [95% CI 4.77−47.16], p < 0.01). There was no influence of VR use on pain or drug consumption. |
| Roxburgh et al. (2021, France) (123) | 99 (48/51) | | Observational study | 3 | Patients | 63 ± 10.9 | n/a | Intraprocedural | 1 of 5 3D computer-simulated scenarios during atrial fibrillation ablation | Standard treatment | Compared to the control group, the mean perceived pain, assessed with the visual analog scale, was lower in the VR group (3.5 [SD 1.5] vs 4.3 [SD 1.6]; P=.004), and comfort was higher in the VR group (7.5 [SD 1.6] vs 6.8 [SD 1.7]; P=.03). Morphine consumption was not different between the groups. Complications, procedure and fluoroscopy duration, were not different between the two groups. |
| Chang et al. (2021, Taiwan) (124) | 52 (52/0) | | Observational study | 3 | Medical staff and patients | 23–30 / 35–54 | 5:20 min | Education | VR informative aids details of the environment, teams, process of disinfection, local anesthesia, insert the sheath, place the puncture  needle in the atrium, and go through the steps of either radiofrequency-ablation | n/a | In addition to the high level of satisfaction, self-efficacy and knowledge scores of residents and their patients were increased after VR aids-based training and resident-led patient education. Higher degree of improvement in self-efficacy was noted among patients with depression/anxiety history than those without history. The positive effects of VR aids were sustained until 2 weeks later. |
| Balsam et al. (2019, Poland) (125) | 100 (100/0) | | Observational study | 3 | Patients | 63 ± 15 | n/a | Patient education | VR for teaching patients about the consequences of atrial fibrillation (AF) and pharmacological stroke prevention | n/a | Before the projection 22/100 declared stroke a consequence of AF, while immediately after 83/100 (p < 0.0001). Seven days after, stroke as AF consequence was chosen by 74/94 (78.7%) vs. 22/94 (23.4%) when compared to the baseline; p < 0.0001, a similar trend was also observed in 1-year follow-up (64/90 [71.1%] vs. 21/90 [23.3%]; p < 0.0001). Before the projection 88.3% (83/94) patients responded, that drugs may reduce the risk of stroke, and after 1 week the number of patients increased to (94/94 [100%]; p = 0.001). After 1 year 87/90 (96.7%) answered that drugs may diminish the risk of stroke (p = 0.02 in comparison to the baseline survey 78/90 [86.7%]). Use of oral anticoagulation to reduce the risk of stroke was initially chosen by 66/94 (70.2%), by 90/94 (95.7%; p < 0.0001) 7 days after and by 83/90 (92.2%; p < 0.0001) 1 year after. |
| Southworth et al. (2020, United states) (126) | 10 (10/10) | | Observational study | 3 | Medical staff | n/a | n/a | Intraprocedural | XR display for guidance during transcatheter cardiac mapping and ablation | Physician feedback | The MXR system achieved acceptable frame rate, latency, and battery runtime with acceptable dynamic range and depth distortion as well as minimal geometric distortion. |
| Prakosa et al. (2021, United states) (127) | 106 (53/53) | | Observational study | 3 | Medical staff | n/a | n/a | Training | AR catheter navigation in virtual ventricular tachycardia (VT) ablations | Standard care | Smaller error with AR significantly resulted in more VT termination (25) compared to the error without AR (16) (P < 0.01). In 52 VT 2 ablation simulations, no significant difference was observed from error with (11) and without AR (13) (P = 0.58). |
| Silva et al. (2020, United states) (128) | 3 (3/0) | | Observational study | 3 | Medical staff | n/a | n/a | Intraprocedural | XR based 3D display of patient specific data during minimally invasive cardiac  electrophysiology procedures | Standard care | The physicians found the system comfortable and easy to use with readily accessible tools. Most physicians (93%) used all the features in the interface and 83% found that the ability to control or manipulate the data was the most important feature.  93% of physicians found that data, when presented in true 3-dimensions and the ability to control the angle of viewing of the data, were easier to interpret than current standard.  87% of physicians found that they learned something new about the anatomy when viewing the data in 3D. |
| Jang et al. (2018, United states) (129) | 5 (5/0) | | Observational study | 3 | Medical staff | n/a | n/a | Preprocedural | 3D holographic visualization of high-resolution myocardial scar | n/a | Operator and mapping specialist found it useful (usefulness rating: operator, 5.8; mapping specialist, 5.5; 1-7 scale) to have scar information during the intervention. HoloLens provides a true 3D perception of the complex scar architecture. |
| Serfözö et al. (2023, Switzerland) (130) | 50 (50/50) | | Observational study | 3 | Medical staff | 66.2 ± 10.2 | n/a | Postprocedural | AR–based method to assess precordial electrocardiogram leads | Standard recording | 86% of the ECGs were assigned correctly, indicating the remarkable similarity of  the smartwatch to standard ECG recordings. |
| James et al. (2020, United States) (131) | 8 (8/8) | | Observational study | 3 | Medical staff | n/a | n/a | Training | VR guided transseptal puncture | Fluoroscopy-guided transseptal puncture | VR guidance resulted in significantly more accurate puncture site selection and was more intuitive for the operator, particularly for novices. |
| Bloom et al. (2023, United States) (132) | 30 (10/20) | | Case-controlled analysis | 3 | Medical staff | n/a | n/a | Intraprocedural | XR system for navigational mapping during cardiac electrophysiological testing | n/a | No statistical difference in total case times between intervention group vs control group (118 ± 29 minutes vs 97 ± 20 minutes; P = .07) or fluoroscopy times (6 ± 4 minutes vs 7 ± 6 minutes; P = .9). No significant difference in case duration (121 ± 26 minutes vs 118 ± 29 minutes; P = .96). During creation of a single cardiac geometry, no significant time difference was noted (284 ± 45 seconds vs 268 ± 43 seconds; P = .1) or fluoroscopy use (9 ± 19 seconds vs 6 ± 18 seconds; P = .25). During point navigation tasks, there was no difference in total time 31 ± 14 seconds vs 28 ± 15 seconds; P = .16) or fluoroscopy time. |
| Silva et al. (2020, United states) (133) | 1 (1/0) | | Case report | 4 | Medical staff | n/a | n/a | Intraprocedural | XR display during cardiac ablation  procedures | n/a | Intraprocedural use of MXR is feasible and will provide a more intuitive visualization |
| Knecht et al. (2018, Switzerland) (134) | 1 (1/0) | | Case report | 4 | Medical staff and engineers | n/a | n/a | Preprocedural planning | VR visualization before ablation in patient with CHD | n/a | The main advantage of VR technology was the ability to follow the access route of the catheter in order to control the catheter manipulation. It is  almost immediately availability (within 30 seconds) without need for  segmentation. |
| Lodzinski et al. (2018, Poland) (135) | 1 (1/0) | | Case report | 4 | Medical staff | n/a | n/a | Intraprocedural | AR for percutaneous pulmonary vein  isolation | n/a | Additional 3D imaging options may be successfully used during electrophysiological procedures to improve the visualization of anatomical structures. |
| Rivero et al. (2024, United States) (136) | 1 (1/1) | | Case report | 4 | Patient | 60 | 240 min | Intraprocedural | VR s an anesthetic alternative for ablation of noninducible ventricular arrhythmia | Standard card | VR could provide a solution to the challenge of balancing patient comfort and optimal conditions for arrhythmia induction and mapping in patients intolerant of awake procedures with local anesthetic alone during ablation of ventricular arrhythmias. |
| **Cardiac Resynchronization Therapy Devices (N=9)** | | | | | | | | | | | |
| Maytin et al. (2015, United states) (137) | 8 (4/4) | | RCT | 2 | Medical staff | 38 ± 1 | 4 h | Training | VR simulator for transvenous lead extraction | Standard training | The VR group executed patient preparation and procedure performance better than the control group (P < 0.01). All fellows in control group experienced a simulator complication (two superior vena cava tears, three right ventricle avulsions) versus one fellow in the VR group (one SVC tear) (P = 0.02). Tactile measurements revealed a trend toward excess pushing versus pulling forces among the conventionally trained group. The time for lead removal was also significantly higher in the control group  (12.46 minutes vs 5.54 minutes, P = 0.02). There was no significant difference in baseline or posttraining cognitive ability. |
| Squara et al. (2024, France) (138) | 61 (30/31) | | RCT | 2 | Patients | 78.3±8.4 / 77.5±8.0 | 30 min | Preprocedural | Premedication and standard analgesia care and 360° video recordings with different static landscapes:  river delta, rural India, Spitzberg, mountains in summer,  or mountains in winter | Premedication and standard analgesia care | Pain and anxiety were lower in the VR-Group during deep venous puncture (3.0 ± 2.0 vs. 4.8 ± 2.2, p = 0.002 and 2.4 ± 2.2 vs. 4.1 ± 2.4, p = 0.006) but not during pocket creation (p = 0.58 and p = 0.5). Morphine consumption was lower in the VR-Group (1.6 ± 0.7 vs. 2.1 ± 1.1 mg; p = 0.041). Patients' overall comfort during procedure was similar in both groups. |
| Drozdova et al. (2024, Czech Republic) (139) | 150 (75/75) | | RCT | 2 | Patients | 77 (68–84) / 76 (70–81) | 6 min | Education | 360° VR video about the pacemaker implantation | Education by a physician | There was no significant difference in the quality of education. There was a non-significant trend towards higher educational scores in the VR group. The subgroup with worse scores was older than the groups with higher scores (82 vs. 76 years, p = 0.025). Anxiety was reduced in 92% of participants. |
| Wong et al. (2001, England) (140) | 32 (32/0) | | Observational study | 3 | Medical staff | n/a | n/a | Training | VR simulator (VRS) for permanent pacing implantation | n/a | VRS does reflect operator experience in pacing system implantation; experienced group has shorter procedure and fluoroscopy time |
| Mascheroni et al. (2020, Ireland) (141) | 26 (26/0) | | Observational study | 3 | Medical staff | n/a | n/a | Training | VR simulator for CRT device implantation | n/a | The procedure metrics used in this study reliably distinguish novice and experienced CRT implanters' performances. The metrics further differentiated performance levels within a group with similar experience. These performance metrics will underpin quality-assured novice implanter training. |
| Carretero et al. (2024, Spain) (142) | 10 (10/0) | | Observational study | 3 | Medical staff | n/a | n/a | Preprocedural | Planning of transvenous lead extraction | n/a | VR seems an estimable aid for operators in planning difficult cases and also an excellent tool for teaching. |
| Witkowski et al. (2019, Poland) (143) | 1 (1/0) | | Case report | 4 | Medical staff | 68 | n/a | Intraprocedural | AR use during CRT device implantation | n/a | Use of AR 3D reconstruction may improve the efficacy of CRT implantation, shorten the procedure time, and allow a reduction of total fluoroscopy dose. |
| Opolski et al. (2018, Poland)(144) | 1 (1/0) | | Case report | 4 | Medical staff | 30 | n/a | Intraprocedural | AR/CT -guided transcatheter pacemaker implantation in dextrocardia and congenitally corrected transposition of great arteries | n/a | AR may optimize complex percutaneous interventions in patients with altered fluoroscopic orientation. |
| Higaki et al.  (2021, Japan) (145) | 1 (1/0) | | Proof-of-concept study | n/a | Medical staff | 65 | n/a | Postprocedural | VR visualization of lead trajectory | n/a | The lead trajectory can be reconstructed from bidirectional radiographs, which may allow for further investigation of the 3D shape change of the pacemaker leads. |
| **Cardiac rehabilitation (N=19)** | | | | | | | | | | | |
| Jozwik et al. (2021, Poland) (146) | 43 (17/26) | | RCT | 2 | Patients | 65.4 ± 8 | 8 therapeutic sessions, 20 min long | Cardiac rehabilitation (CR) | CR + VR session with a virtual therapy garden | CR + Schultz autogenic training sessions | In the VR group, the sole parameter which failed to improve was HADS-Anxiety. In the control group, there was a deterioration in nearly all tested parameters except for HADS-Depression. Statistically significant differences in the efficacy of rehabilitation were recorded in relation to the level of stress in the sub-scales: emotional tension (p = 0.005), external stress (p = 0.012), intrapsychic stress (p = 0.023) and the generalized stress scale (p = 0.004). |
| Jozwik et al. (2021, Poland) (147) | 77 (28/49) | | RCT | 2 | Patients | 64.7 ± 8.03 | 8 therapeutic sessions, 20 min long | CR | CR + VR session with a virtual therapy garden | CR + Schultz autogenic training sessions | In the VR group, the overall HADS score was statistically significantly reduced by 13.5%, HADS-Depression by 20.8%, and the general stress level by 12.8% (p < 0.05). In the control group, the scores were statistically significantly higher. |
| Gulick et al. (2021, United states) (148) | 72 (41/31) | | RCT | 2 | Patients | 32-81 | Up to 15 minutes | CR | VR walking trails, which included audio-recorded education | Standard rehabilitation | 6-minute walk test, no significant differences were observed between the intervention and control groups (P=.64). No statistical differences were observed between groups in terms of education (P=.86) or satisfaction (P=.32). The control group had statistically more favorable rates of attendance, as determined by the risk group comparison (P=.02) and the comparison of the rates for completing the minimum number of sessions (P=.046), but no correlation was observed between the study group and reasons for ending treatment. |
| Szczepanska-Gieracha et al. (2021, Poland) (149) | 32 (15/17) | | RCT | 2 | Patients | 68.91 ± 6.26 | 8 therapeutic sessions, 20 min long | CR | CR + VR session with a virtual therapy garden | CR + Schultz autogenic training sessions | In the VR group, a significant decrease in HADS score was observed (19.46 pretreatment vs. 15.73 post-treatment, p = 0.003), HADS-Anxiety subscale decreased by 16% (p = 0.01) and HADS-Depression by 23 % (p = 0.003). Similarly, a significant decrease in PSQ was recorded at 12.8 % (64.73 vs. 56.47, p = 0.03). In the control group, HADS and PSQ data did not change. |
| Garcia-Bravo et al. (2020, Spain) (150) | 20 (10/10) | | RCT | 2 | Patients | 51.20 ±8.82 | 16 sessions, 20 min long | CR | Training based on VR of aerobic exercise using the XBOX ONE console and Kinect sensor | Standard rehabilitation | VR-based video game program showed improvements in ergometry, METS, resistance to fatigue, and health-related quality of life with excellent adherence and satisfaction perceived by patients. |
| Maciolek et al. (2020, Poland) (151) | 65 (32/33) | | RCT | 2 | Patients | 59.8 ± 11.8 | 6 session, 20 min long | CR | VR relaxation training with a natural virtual land‑  scape model accompanied by relaxation music | Relaxation training  conducted  by a psychologist | After the treatment, there was a reduction in the mean intensity of the anxiety‑trait indicator in both  Groups. There was a reduction in the mean anxiety‑state in the VR group (P = 0.01) as opposed to the control group (P = 0.38). |
| Da Cruz et al. (2021, Brasil) (152) | 61 (30/31) | | RCT | 2 | Patients | 63.27 ± 12.68 | One session in a week for 12 weeks | CR | In the VR session, the warm-up was performed using "Just Dance 2015" game, in which patients reproduced choreography in a previously defined sequence. The exercise training phase was performed through "Shape Up" exercise games following a virtual therapist. | Standard rehabilitation | CR + VR resulted in a significant increase in adherence at 12 weeks (baseline = 72.87%; 12 weeks = 82.80%), with significant reductions at 24 weeks when VR was withdrawn (65.48%); in the control group, there were no changes over time. Motivation decreased significantly from baseline to 12 weeks (4.32 [SD = 0.37] vs 4.02 [SD = 0.76]) and significantly increased from 12 to 24 weeks in the CR + VR group (4.37 [SD = 0.36]). Absorption was significantly lower at 12 weeks in the VR group (6.79 [SD = 0.37] vs 6.20 [SD = 1.01]). |
| Vieira et al. (2018, Portugal) (153) | 33 (11/11/11) | | RCT | 2 | Patients | 55 ± 9 | n/a | CR | VR game system, using the Kinect sensor | Paper booklet or usual care | The VR format had improved selective attention and conflict resolution ability, revealing the potential of CR, specifically with virtual reality exercise, on executive function. No significant differences were found in the quality of life, and depression, anxiety and stress. |
| Yuenyongchaiwat et al. (2024, Thailand) (154) | 60 (30/30) | | RCT | 2 | Patients | 63.20 ± 9.57 /  64.43 ± 8.74 | 10 min daily until discharge from the hospital | CR | VR exercise program | Conventional physical therapy | In comparison, there was no significant increase in cardiopulmonary performance between the two groups. the conventional physical therapy group showed significantly higher depression scores than the VR group (∆4.00 ± 0.98 vs. ∆1.68 ± 0.92). |
| Vieira et al. (2017, Portugal) (155) | 33 (11/11/11) | | RCT | 2 | Patients | 55 ± 9.0 / 59 ± 11.3 / 59  ± 5.8 | n/a | CR | VR game system, using the Kinect sensor | Paper booklet or usual care | The VR group revealed significant improvements in the waist-to-hip ratio after 6 months (p= 0.033) and, between the baseline and third month, when compared  with the control  group (p= 0.041).The VR group decreased  their ingestion  of total fat (p=  0.032) after six  months and increased the high-density lipoprotein cholesterol  (p=0.017) 3 months after the program’s conclusion. |
| Wrzeciono et al. (2024, Poland) (156) | 50 (25/25) | | RCT | 2 | Patients | 59.40 ± 5.56 / 72.72 ± 5.37 | 8–10 therapeutic sessions lasting 20 min per session | CR | VR therapy to cope with anxiety and depression for younger (<65 years) and older (>65 years) patients | n/a | It appears that older patients benefit equally from psychological intervention in a virtual setting as younger patients. Age is not a factor that predicts the effectiveness of VR therapy in reducing anxiety and depression in patients with cardiovascular disease. |
| Da Cruz et al. (2020, Brazil) (157) | 27 (27/27) | | Cluster-randomized crossover trial | 3 | Patients | 63.4 ±  12.7 | One session in a week for 12 weeks | CR | In the VR session, the warm-up was performed using "Just Dance 2015" game, in which patients reproduced choreography in a previously defined sequence. The exercise training phase was performed through "Shape Up" exercise games following a virtual therapist. | Standard rehabilitation | VR produces a physiological similar pattern of acute hemodynamic effects in CR. There was greater magnitude of heart rate, respiratory rate, and rating of perceived exertion (P<.01) during the execution of VR training. |
| Silva et al. (2021, Brazil) (158) | 28 (28/28) | | Cluster-randomized crossover trial | 3 | Patients | 63.39 ± 12.48 | One session in a week for 12 weeks | CR | In the VR session, the warm-up was performed using "Just Dance 2015" game, in which patients reproduced choreography in a previously defined sequence. The exercise training phase was performed through "Shape Up" exercise games following a virtual therapist. | Standard rehabilitation | Cardiac autonomic modulation during and after the VR was comparable to standard, yet, the extents were greater in the VR group. After 12 weeks of VR training, the subjects adapted to the exercises from the 15th minute and exhibited faster recovery compared to the 1st week. |
| Da Cruz et al. (2022, Brasil) (159) | 26 (26/0) | | Observational study | 3 | Patients | 62.04 ± 12.22 | n/a | CR | VR-based therapy | n/a | The combination of VR as routine in a traditional program did not cause significant changes in the analyzed variables: systolic and diastolic blood pressure, respiratory rate, pulse saturation of oxygen, heart rate, perceived exertion, and cardiac autonomic modulation. |
| Vorwerg-Gall et al. (2024, Germany) (160) | 12 (12/12/12) | | Observational study | 3 | Patients | 74.42 ± 5.84 | 5 min | CR | MR-based remote workout with a holographic trainer | Workout-Group (WG) (the participants were represented as gamified avatars on a screen) or Screen (the participants could see themselves and the trainer as a point cloud on a screen) | No significant differences in heart rate were found between the three sessions. The participants rated the usability on the system usability scale as good and had a high level of enjoyment in all physical activities. WG was favored by the older adults with hypertension (n = 9) and would be the most preferred by them in a future home setting (n = 6). |
| Jozwik et al. (2022, Poland) (161) | 34 (17/17) | | Observational study | 3 | Patients | 63.82 ± 8.13 | 8 therapeutic sessions, 20 min long | CR | CR + VR session with a virtual therapy garden, where the patient was supposed to calm down and relax | CR + Schultz autogenic training sessions | Improvement in the mental state of the patients and thus has a positive effect on the course of cardiac rehabilitation |
| Da Cruz et al. (2022, Brasil) (162) | 15 (15/0) | | Observational study | 3 | Patients | 60.80 ±  14.51 | One session in a week for 12 weeks | CR | In the VR session, the warm-up was performed using "Just Dance 2015" game, in which patients reproduced choreography in a previously  defined sequence. The exercise training phase was performed through "Shape Up" exercise games following a virtual therapist. | Standard rehabilitation | Good acceptance and satisfaction in VR group. Physical and psychosocial benefits were highlighted, and patients reported the perception of higher exercise intensity with VR then when doing conventional training. Cognitive aspects that influenced participation to the new approach were also raised. |
| Vieira et al. (2018, Portugal) (163) | 11 (11/0) | | Observational study | 3 | Patients | 55 | 3 sessions per week | CR | VR game system, using the Kinect sensor | n/a | 91% of the participants (n = 10) enjoyed the artwork, while 100% (n = 11) agreed on the importance and usefulness of the automatic counting of the number of repetitions. 64% (n = 7) reported motivation to continue performing the programme after the end of the study, and 100% (n = 11) recognized Kinect as an instrument with potential to be an  asset in CR Criticisms included limitations in motion capture and gesture recognition, 91%  (n = 10), and the lack of home space, 27% (n = 3). |
| Klompstra et al. (2013, Sweden) (164) | 1 (1/0) | | Case report | 4 | Patients | 74 | 15 min every day for 12 weeks | CR | VR system using Nintendo Wii | n/a | Playing the Nintendo Wii did not increase the perceived physical effort, but increased motivation to exercise and decreased barriers to exercising. |

Table S2: Overview of XR clinical trials

| **ClinicalTrials.gov ID** | **Title** | **Study status** | **Location** |
| --- | --- | --- | --- |
| NCT05938218 | Virtual Reality Assisted Patient Empowerment: Diagnose ATTR-Amyloidosis And Start Treatment (VRAP-DAAST) | NOT YET RECRUITING | Germany |
| NCT04984655 | Virtual Reality Experience for Stress Reduction in Cardiology Patients | COMPLETED | United States |
| NCT05588232 | The Effectiveness of Therapeutic Virtual Reality Versus Pharmacological Sedation on Pain and Anxiety During Interventional Cardiology Procedures (VirtuCardio) | COMPLETED | France |
| NCT04736888 | Effectiveness of Extended Reality CPR Training Methods | RECRUITING | Republic of Korea |
| NCT05798832 | An Evaluation of Virtual Reality and Traditional Training in Basic Life Support Training (VR-BLS) | COMPLETED | Turkey |
| NCT06346132 | Effect of Virtual Reality on Patient's Comfort During Cardiac Electronic Device Implantation. | NOT YET RECRUITING | Switzerland |
| NCT03490903 | Coronary Angiography THerapeutic Virtual Reality (CATH-VR) | WITHDRAWN | United States |
| NCT06445387 | Pain During Femoral Sheath Removal in Patients Undergoing Percutaneous Coronary Intervenion | COMPLETED | Turkey |
| NCT06288087 | Comparing Machine Guided VR Based Training With Educator Guided Training in Metaverse | RECRUTING | Turkey |
| NCT04313777 | Virtual Reality Therapy in Cardiology | COMPLETED | Poland |
| NCT03350971 | Influence of Virtual Reality on Myocardial Revascularization | COMPLETED | Brazil |
| NCT05069987 | Anxiety Reduction in TAVI Using Virtual Reality Trial (ART-VR) | UNKNOWN STATUS | Netherlands |
| NCT04242563 | Virtual Reality for Preoperative Anxiety in Interventional Cardiology (No PANIC) | COMPLETED | France |
| NCT04259723 | Virtual Reality Assisted Conscious Sedation During TAVI (TAVI_VR) | COMPLETED | Germany |
| NCT04336306 | Virtual Reality-based Therapy and Barriers in Cardiac Patients | COMPLETED | Brazil |
| NCT03945201 | Virtual Reality-enhanced Exercise and Education in Cardiac Rehabilitation | COMPLETED | United States |
| NCT02949401 | Stress Inoculation Through Virtual Reality in the Pediatric Electrophysiology Laboratory | COMPLETED | United States |
| NCT03377582 | Virtual Reality Based-therapy Applied to Physical Therapy in Cardiology. | COMPLETED | Brazil |
| NCT04166422 | Virtual Reality and Video Games in Cardiac Rehabilitation Programs (VirtualCORE) | UNKNOWN STATUS | Spain |
| NCT06215456 | Virtual Reality to Reduce Periprocedural Anxiety During Invasive Coronary Angiography (VR InCard) | RECRUITING | Netherlands |
| NCT06458452 | Enhancing Immersion in Virtual Reality Based Advanced Life Support Training | RECRUITING | Turkey |
| NCT02711631 | Feasibility and Effectiveness of Remote Virtual Reality-Based Cardiac Rehabilitation | COMPLETED | Canada |
| NCT04521660 | The Effect of Using Virtual Reality Glasses During Coronary Angiography | UNKNOWN STATUS | Turkey |
| NCT06115928 | Effects of Virtual Reality on Rehabilitation in Patients With Heart Failure (VIRTUAL-HF) | NOT YET RECRUITING | Italy |
| NCT06458647 | The Effect of Video Streaming With Virtual Reality Before Coronary Angiography | NOT YET RECRUITING | Turkey |
| NCT05695547 | VR Education in Patients Undergoing Coronary Angiography | NOT YET RECRUITING | Czechia |
| NCT04944667 | Use of Virtual Reality for Procedural Planning of Transcatheter Aortic Valve Replacement | COMPLETED | Spain |
| NCT04045977 | Virtual Therapy as a Method Supporting the Cardiac Rehabilitation | COMPLETED | Poland |
| NCT06171620 | Virtual Sedation During Elective Procedures in the Cathlab | RECRUITING | Belgium |
| NCT05695534 | Use of VR in Patients' Education Prior to Implantation of PPM | NOT YET RECRUITING | Czechia |
| NCT03662607 | Impact of Virtual Reality on Pre-procedural anxieTy Prior to Heart cathEterIzAtion - VR-THEIA (VR-THEIA) | ACTIVE, NOT RECRUITING | United States |
| NCT05458999 | Decreasing Patient Anxiety During Revascularization of Chronic Total Coronary Occlusions Using Virtual Reality Glasses. (ReViCTO) | UNKNOWN STATUS | Spain |
| NCT05552352 | VRAP-Heart - Virtual Reality Assisted Patient Empowerment for Interventions in Structural Heart Disease (VRAP-Heart) | RECRUITING | Germany |
| NCT05735808 | VR Education in Arterial Hypertension | RECRUITING | Czechia |
| NCT03169387 | Body Composition of Individuals With Cardiovascular Diseases | UNKONOWN STATUS | Brazil |
| NCT04013633 | Lowlands Saves Lives: A Randomized Trial Comparing CPR-quality Between Face-to-face vs. Lifesaver VR Training | COMPLETED | Netherlands |
| NCT04561596 | Virtually Augmented Self Hypnosis in Peripheral Vascular Interventions | COMPLETED | Switzerland |
| NCT06376643 | Augmented Reality to Support Cardiopulmonary Resuscitation | NOT YET RECRUITING | Canada |
| NCT05063773 | Novel Wireless Mixed Reality Headset for Image Guidance in Cardiac Catheterization Laboratory (MRCCL) | UNKONOWN STATUS | United States |
| NCT06057285 | Efficacy of an Augmented Reality Application Developed for ACLs Training on the Knowledge and Skill Levels of Nurses | COMPLETED | Taiwan |
| NCT03531424 | Augmented-Reality CTA Plus Angiography vs Angiography Alone for Guiding PCI in Coronary Lesions - Randomized Study (AR-PCI) | UNKNOWN STATUS | Netherlands |
| NCT04736888 | Effectiveness of Extended Reality CPR Training Methods | RECRUITING | Republic of Korea |
| NCT06539416 | Mixed Reality in MitraClip Implantation: Preliminary Case Series (MIMIC) | NOT YET RECRUITING | Czechia |

**References**

1. Bruno RR, Lin Y, Wolff G, Polzin A, Veulemans V, Klein K, et al. Virtual reality-assisted conscious sedation during transcatheter aortic valve implantation: a randomised pilot study. EuroIntervention. 2020;16(12):e1014-e20.

2. Lind A, Ahsan M, Totzeck M, Al-Rashid F, Haddad A, Dubler S, et al. Virtual reality-assisted distraction during transcatheter aortic valve implantation under local anaesthesia: A randomised study. Int J Cardiol. 2023;387:131130.

3. Vanhoorebeeck F, Breebaart MB, Maes S, Mertens P. Pitfalls and benefits of virtual reality hypnosis during transcatheter aortic valve implantation in high risk patients. Acta Anaesthesiologica Belgica. 2021;72:281-6.

4. Ruyra X, Permanyer E, Huguet M, Maldonado G. Use of virtual reality for procedural planning of transcatheter aortic valve replacement. Interact Cardiovasc Thorac Surg. 2022;35(5).

5. Carnahan P, Moore J, Bainbridge D, Wheeler G, Deng S, Pushparajah K, et al. Applications of VR medical image visualization to chordal length measurements for cardiac procedures2020. 73 p.

6. Narang A, Hitschrich N, Mor-Avi V, Schreckenberg M, Schummers G, Tiemann K, et al. Virtual Reality Analysis of Three-Dimensional Echocardiographic and Cardiac Computed Tomographic Data Sets. J Am Soc Echocardiogr. 2020;33(11):1306-15.

7. Kanschik D, Haschemi J, Heidari H, Klein K, Afzal S, Maier O, et al. Feasibility, Accuracy, and Reproducibility of Aortic Valve Sizing for Transcatheter Aortic Valve Implantation Using Virtual Reality. J Am Heart Assoc. 2024;13(15):e034086.

8. Jolley MA, Lasso A, Nam HH, Dinh PV, Scanlan AB, Nguyen AV, et al. Toward predictive modeling of catheter-based pulmonary valve replacement into native right ventricular outflow tracts. Catheter Cardiovasc Interv. 2019;93(3):E143-e52.

9. van den Bosch AE, Koning AH, Meijboom FJ, McGhie JS, Simoons ML, van der Spek PJ, et al. Dynamic 3D echocardiography in virtual reality. Cardiovasc Ultrasound. 2005;3:37.

10. Kamiya K, Matsubayashi Y, Terada S, Nagatani Y, Fujii T, Nakata S, et al. Ex-vivo aortic root and coronary artery cast measurement to validate the accuracy of virtual imaging. J Card Surg. 2022;37(8):2461-5.

11. Kamiya K, Nagatani Y, Matsubayashi Y, Mori Y, Wakisaka H, Lee J, et al. A Virtual-Reality Imaging Analysis of the Dynamic Aortic Root Anatomy. Ann Thorac Surg. 2021;112(6):2077-83.

12. Kamiya K, Nagatani Y, Terada S, Matsubayashi Y, Minamidate N, Takashima N, et al. Validation of Virtual Imaging of a Dynamic, Functioning Aortic Valve Using an Ex Vivo Porcine Heart. Ann Thorac Surg. 2022;114(1):334-9.

13. Currie ME, McLeod AJ, Moore JT, Chu MW, Patel R, Kiaii B, et al. Augmented Reality System for Ultrasound Guidance of Transcatheter Aortic Valve Implantation. Innovations (Phila). 2016;11(1):31-9; discussion 9.

14. Chahine J, Mascarenhas L, Yannopoulos D, Raveendran G, Gurevich S. Virtual reality to predict paravalvular leak in bicuspid severe aortic valve stenosis in transcatheter aortic valve implants. J Invasive Cardiol. 2024.

15. Zablah JE, Than J, Browne LP, Rodriguez S, Morgan GJ. Patient Screening for Self-Expanding Percutaneous Pulmonary Valves Using Virtual Reality. J Am Heart Assoc. 2024;13(6):e033239.

16. Castellanos JM, Yefimov A, Dang PN. 360-Degree Virtual Reality Consultation for the Structural Heart Disease Patient. Structural Heart-the Journal of the Heart Team. 2020;4(3):230-5.

17. Bonanni M, Russo G, De Siati M, Tomao F, Massaro G, Benedetto D, et al. Holographic mixed reality for planning transcatheter aortic valve replacement. Int J Cardiol. 2024;412:132330.

18. d'Aiello AF, Schianchi L, Bevilacqua F, Ferrero P, Micheletti A, Negura DG, et al. Holography-guided procedural planning for modifying Venus P-valve implantation technique in patients with left pulmonary artery stents: a case-series. Front Cardiovasc Med. 2024;11:1378924.

19. Van De Bruaene A, De Buck S, Verbrugghe P, Dubois C. Preprocedural imaging for transcatheter mitral valve-in-valve replacement: Planning makes perfect. Clin Case Rep. 2022;10(2):e05392.

20. Sacha J, Krawczyk K, Bugajski J, Stanisz M, Feusette P, Gierlotka M. MitraClip Implantation in Holography. JACC Cardiovasc Interv. 2022;15(9):e107-e8.

21. Kasprzak JD, Pawlowski J, Peruga JZ, Kaminski J, Lipiec P. First-in-man experience with real-time holographic mixed reality display of three-dimensional echocardiography during structural intervention: balloon mitral commissurotomy. Eur Heart J. 2020;41(6):801.

22. Castellanos JM, Barbery D, Yefimov A, Dang PN. Preoperative planning using virtual reality for percutaneous transseptal valve-in-valve transcatheter mitral valve replacement: a case report. Eur Heart J Case Rep. 2022;6(10):ytac384.

23. Sadri S, Loeb GJ, Grinshpoon A, Elvezio C, Sun SH, Ng VG, et al. First Experience With Augmented Reality Guidance for Cerebral Embolic Protection During TAVR. JACC Adv. 2024;3(3):100839.

24. Pool MDO, Hooglugt JLQ, Kraaijeveld AJ, Mulder BJM, de Winter RJ, Schijven MP, et al. Pre-procedural virtual reality education reduces anxiety in patients undergoing atrial septal closure - Results from a randomized trial. International Journal of Cardiology Congenital Heart Disease. 2022;7.

25. Heidari H, Kanschik D, Erkens R, Maier O, Wolff G, Bruno RR, et al. Left atrial appendage sizing for percutaneous closure in virtual reality—a feasibility study. Frontiers in Cardiovascular Medicine. 2023;10.

26. Pasquali M, Fusini L, Italiano G, Maltagliati A, Tamborini G, Penso M, et al. Feasibility study of a mixed reality tool for real 3D visualization and planning of left atrial appendage occlusion. J Cardiovasc Comput Tomogr. 2022;16(5):460-2.

27. Mill J, Montoliu H, Moustafa AH, Olivares AL, Albors C, Aguado AM, et al. Domain expert evaluation of advanced visual computing solutions and 3D printing for the planning of the left atrial appendage occluder interventions. Int J Bioprint. 2023;9(1):640.

28. Tejman-Yarden S, Freidin D, Nagar N, Parmet Y, Abed M, Vazhgovsky O, et al. Virtual reality utilization for left atrial appendage occluder device size prediction. Heliyon. 2023;9(4):e14790.

29. Heidari H, Kanschik D, Maier O, Wolff G, Brockmeyer M, Masyuk M, et al. A comparison of conventional and advanced 3D imaging techniques for percutaneous left atrial appendage closure. Front Cardiovasc Med. 2024;11:1328906.

30. Sadeghi AH, Ooms JF, Bakhuis W, Taverne Y, Van Mieghem NM, Bogers A. Immersive Virtual Reality Heart Models for Planning of Transcatheter Paravalvular Leak Closure: A Feasibility Study. JACC Cardiovasc Interv. 2021;14(16):1854-6.

31. Vanreusel I, Maes S, De Wolf D, Van Berendoncks A. Percutaneous closure of a secundum atrial septum defect performed under hypnosis: a case report. Acta Cardiol. 2021;76(10):1078-82.

32. Shimura T, Higami H, Matsuo H, Yamamoto M. Appropriate assessment using virtual reality simulation for a novel reshaped curve sheath during percutaneous left atrial appendage closure: a follow-up case report. Eur Heart J Case Rep. 2023;7(11):ytad503.

33. Zbroński K, Rymuza B, Scisło P, Kochman J, Huczek Z. Augmented reality in left atrial appendage occlusion. Kardiol Pol. 2018;76(1):212.

34. Złahoda-Huzior A, Januska J, Hecko J, Khokhar A, Dudek D. Virtual reality-assisted heart team consultation for complex structural heart intervention. Eur Heart J Case Rep. 2023;7(1):ytac477.

35. Sawada F, Yamamoto M, Shimura T, Higami H. Successful percutaneous left atrial appendage closure using reshaped curve sheath created by simultaneous virtual reality simulator. Eur Heart J Case Rep. 2024;8(8):ytae399.

36. Patel N, Costa A, Sanders SP, Ezon D. Stereoscopic virtual reality does not improve knowledge acquisition of congenital heart disease. Int J Cardiovasc Imaging. 2021;37(7):2283-90.

37. Wang LW, Liu JF, Xie WP, Chen Q, Cao H. Condition notification assisted by virtual reality technology reduces the anxiety levels of parents of children with simple CHD: a prospective randomised controlled study. Cardiology in the Young. 2022;32(11):1801-6.

38. Kieu V, Sumski C, Cohen S, Reinhardt E, Axelrod DM, Handler SS. The Use of Virtual Reality Learning on Transition Education in Adolescents with Congenital Heart Disease. Pediatr Cardiol. 2023;44(8):1856-60.

39. Bertelli F, Raimondi F, Godard C, Bergonzoni E, Cattapan C, Gastino E, et al. Fast-track virtual reality software to facilitate 3-dimensional reconstruction in congenital heart disease. Interdiscip Cardiovasc Thorac Surg. 2023;36(6).

40. Awori J, Friedman SD, Howard C, Kronmal R, Buddhe S. Comparative effectiveness of virtual reality (VR) vs 3D printed models of congenital heart disease in resident and nurse practitioner educational experience. 3D Print Med. 2023;9(1):2.

41. Vegulla RV, Tandon A, Rathinaswamy J, Cherian KM, Hussain T, Murala JS. Advanced imaging and digitization of preserved heart specimens using virtual reality - A primer. Ann Pediatr Cardiol. 2022;15(4):351-7.

42. Pajaziti E, Milano EG, Schievano S, Cook A, Capelli C. Investigating the Feasibility of Virtual Reality for Teaching on Congenital Heart Disease. European Journal of Vascular and Endovascular Surgery. 2022;63(2):e42.

43. Sun K, Xue HH, Yu JG, Wang J, Chen GZ, Hong WJ, et al., editors. A primary exploration of three-dimensional echocardiographic intra-cardiac virtual reality visualization of atrial septal defect: In vitro validation. 32nd Annual Conference on Computers in Cardiology; 2005 Sep 25-28; Lyon, FRANCE2005.

44. Topuzov N, Jaszewska A, Migdał A, Brzezińska-Rajszys G, Zubrzycka M, Rewers B, et al. Virtual simulations in planning intravascular treatment of aortic coarctation - a retrospective analysis. Postepy Kardiol Interwencyjnej. 2022;18(3):276-82.

45. Lim TR, Wilson HC, Axelrod DM, Werho DK, Handler SS, Yu S, et al. Virtual reality curriculum increases paediatric residents' knowledge of CHDs. Cardiol Young. 2023;33(3):410-4.

46. Kang SL, Shkumat N, Dragulescu A, Guerra V, Padfield N, Krutikov K, et al. Mixed-reality view of cardiac specimens: a new approach to understanding complex intracardiac congenital lesions. Pediatric Radiology. 2020;50(11):1610-6.

47. Ghosh RM, Jolley MA, Mascio CE, Chen JM, Fuller S, Rome JJ, et al. Clinical 3D modeling to guide pediatric cardiothoracic surgery and intervention using 3D printed anatomic models, computer aided design and virtual reality. 3D Print Med. 2022;8(1):11.

48. d'Aiello AF, Cabitza F, Natali C, Viganò S, Ferrero P, Bognoni L, et al. The Effect of Holographic Heart Models and Mixed Reality for Anatomy Learning in Congenital Heart Disease: An Exploratory Study. J Med Syst. 2023;47(1):64.

49. Xue H, Sun K, Yu J, Chen B, Chen G, Hong W, et al. Three-dimensional echocardiographic virtual endoscopy for the diagnosis of congenital heart disease in children. Int J Cardiovasc Imaging. 2010;26(8):851-9.

50. Lau I, Gupta A, Sun Z. Clinical Value of Virtual Reality versus 3D Printing in Congenital Heart Disease. Biomolecules. 2021;11(6).

51. Kim B, Loke YH, Mass P, Irwin MR, Capeland C, Olivieri L, et al. A Novel Virtual Reality Medical Image Display System for Group Discussions of Congenital Heart Disease: Development and Usability Testing. JMIR Cardio. 2020;4(1):e20633.

52. Lau I, Gupta A, Ihdayhid A, Sun Z. Clinical Applications of Mixed Reality and 3D Printing in Congenital Heart Disease. Biomolecules. 2022;12(11).

53. Giffoni MC, Lopes J, Ribeiro G, Araujo Júnior E, Werner H. Fetal heart segmentation in a virtual reality environment. Int J Cardiovasc Imaging. 2024.

54. Salavitabar A, Dutro M, Zablah JE. Virtual Reality Remote Collaboration for Preprocedural Planning of Complex Percutaneous Congenital Interventions: A Case Series. J Soc Cardiovasc Angiogr Interv. 2024;3(3Part B):101302.

55. Mejia E, Morgan GJ, Stone M, Shorofsky MJ, Jaggers J, Zablah JE. Advanced Imaging Guidance for Ductus Venosus Stenting in Obstructed Total Anomalous Pulmonary Venous Return. World J Pediatr Congenit Heart Surg. 2024:21501351241247513.

56. Tandon A, Burkhardt BEU, Batsis M, Zellers TM, Velasco Forte MN, Valverde I, et al. Sinus Venosus Defects: Anatomic Variants and Transcatheter Closure Feasibility Using Virtual Reality Planning. JACC Cardiovasc Imaging. 2019;12(5):921-4.

57. Kasprzak JD, Witowski J, Pawlowski J, Peruga JZ, Złahoda-Huzior A. Percutaneous patent ductus arteriosus closure using intraprocedural mixed reality visualization of 3D computed tomography angiography data: first-in-man experience. Eur Heart J Cardiovasc Imaging. 2019;20(7):839.

58. Ghosh RM, Mascio CE, Rome JJ, Jolley MA, Whitehead KK. Use of Virtual Reality for Hybrid Closure of Multiple Ventricular Septal Defects. JACC Case Rep. 2021;3(14):1579-83.

59. Sivakumar K, Sagar P, Thejaswi P. Virtual Reality Software Aids Precise Transcatheter Closure of Sinus Venosus Defect Using Covered Stent Without 3D Printing. Pediatr Cardiol. 2024;45(4):939-41.

60. Szeliga J, Lazu M, Góreczny S. Virtual reality modelling in a child after multiple interventions for hypoplastic aortic arch and coarctation. Cardiol Young. 2024:1-3.

61. Wilson HC, Hashemi S, Ligon RA. 4-Dimensional Right Ventricular Outflow Tract Segmentation to Evaluate Candidacy for Transcatheter Pulmonary Valve Replacement. Pediatr Cardiol. 2024.

62. Dziewulska A, Szeliga J, Góreczny S. Virtual reality modelling for planning of percutaneous first step palliation in a newborn with heterotaxy syndrome. Cardiol Young. 2024:1-3.

63. Galeczka M, Smerdzinski S, Tyc F, Fiszer R. Virtual reality for transcatheter procedure planning in congenital heart disease. Kardiol Pol. 2023.

64. Larsson CE, Cabassut V, Peretout P, Marliere S, Vautrin E, Piliero N, et al. Assessment of the Objective Effect of Virtual Reality for Preoperative Anxiety in Interventional Cardiology. Am J Cardiol. 2023;205:207-13.

65. Gökçe E, Arslan S. Effects of virtual reality and acupressure interventions on pain, anxiety, vital signs and comfort in catheter extraction processes for patients undergoing coronary angiography: A randomized controlled trial. Int J Nurs Pract. 2023:e13176.

66. Morgan H, Nana M, Phillips D, Gallagher S. The Effect of a VIrtual RealiTy Immersive Experience Upon Anxiety Levels, Procedural Understanding, and Satisfaction in Patients Undergoing CArdiac CaTHeterization: The VIRTUAL CATH Trial. J Invasive Cardiol. 2021;33(9):E681-e6.

67. Popovic B, Pinelli S, Albuisson E, Metzdorf PA, Mourer B, Tran N, et al. The Simulation Training in Coronary Angiography and Its Impact on Real Life Conduct in the Catheterization Laboratory. Am J Cardiol. 2019;123(8):1208-13.

68. Cates CU, Lönn L, Gallagher AG. Prospective, randomised and blinded comparison of proficiency-based progression full-physics virtual reality simulator training versus invasive vascular experience for learning carotid artery angiography by very experienced operators. BMJ Simul Technol Enhanc Learn. 2016;2(1):1-5.

69. Voelker W, Petri N, Tönissen C, Störk S, Birkemeyer R, Kaiser E, et al. Does Simulation-Based Training Improve Procedural Skills of Beginners in Interventional Cardiology?--A Stratified Randomized Study. J Interv Cardiol. 2016;29(1):75-82.

70. Jensen UJ, Jensen J, Ahlberg G, Tornvall P. Virtual reality training in coronary angiography and its transfer effect to real-life catheterisation lab. EuroIntervention. 2016;11(13):1503-10.

71. Bagai A, O'Brien S, Al Lawati H, Goyal P, Ball W, Grantcharov T, et al. Mentored simulation training improves procedural skills in cardiac catheterization: a randomized, controlled pilot study. Circ Cardiovasc Interv. 2012;5(5):672-9.

72. Aeckersberg G, Gkremoutis A, Schmitz-Rixen T, Kaiser E. The relevance of low-fidelity virtual reality simulators compared with other learning methods in basic endovascular skills training. Journal of Vascular Surgery. 2019;69(1):227-35.

73. Perez-Gutierrez B, Uribe-Quevedo A, Vega-Medina L, Salgado JS, Jaimes N, Perez O, editors. Immersive and Non-Immersive VR Percutaneous Coronary Intervention Simulation for Acute Myocardial Infarction. IEEE 8th International Conference on Serious Games and Applications for Health (SeGAH); 2020 Aug 12-14; Vancouver, CANADA2020.

74. Willaert WI, Aggarwal R, Daruwalla F, Van Herzeele I, Darzi AW, Vermassen FE, et al. Simulated procedure rehearsal is more effective than a preoperative generic warm-up for endovascular procedures. Ann Surg. 2012;255(6):1184-9.

75. Van Herzeele I, Aggarwal R, Choong A, Brightwell R, Vermassen FE, Cheshire NJ. Virtual reality simulation objectively differentiates level of carotid stent experience in experienced interventionalists. J Vasc Surg. 2007;46(5):855-63.

76. Li S, Cui JH, Hao AM, Zhang SY, Zhao QP. Design and Evaluation of Personalized Percutaneous Coronary Intervention Surgery Simulation System. Ieee Transactions on Visualization and Computer Graphics. 2021;27(11):4150-60.

77. Jensen UJ, Jensen J, Olivecrona G, Ahlberg G, Lagerquist B, Tornvall P. The role of a simulator-based course in coronary angiography on performance in real life cath lab. Bmc Medical Education. 2014;14.

78. Aardoom JJ, Hilt AD, Woudenberg T, Chavannes NH, Atsma DE. A Preoperative Virtual Reality App for Patients Scheduled for Cardiac Catheterization: Pre-Post Questionnaire Study Examining Feasibility, Usability, and Acceptability. JMIR Cardio. 2022;6(1):e29473.

79. Nicholson WJ, Cates CU, Patel AD, Niazi K, Palmer S, Helmy T, et al. Face and content validation of virtual reality simulation for carotid angiography: results from the first 100 physicians attending the Emory NeuroAnatomy Carotid Training (ENACT) program. Simul Healthc. 2006;1(3):147-50.

80. Vardhan M, Shi H, Gounley J, Chen SJ, Kahn A, Leopold J, et al., editors. Investigating the Role of VR in a Simulation-Based Medical Planning System for Coronary Interventions. 10th International Workshop on Machine Learning in Medical Imaging (MLMI) / 22nd International Conference on Medical Image Computing and Computer-Assisted Intervention (MICCAI); 2019 Oct 13-17; Shenzhen, PEOPLES R CHINA2019.

81. Räder S, Abildgaard U, Jorgensen E, Bech B, Lönn L, Ringsted CV. Association Between Endovascular Performance in a Simulated Setting and in the Catheterization Laboratory. Simulation in Healthcare-Journal of the Society for Simulation in Healthcare. 2014;9(4):241-8.

82. Popovic B, Varlot J, Girard-Bertrand B, Basile JL, Thouvenot C, Fay R, et al. Impact of Simulation-Based Training on Radiation Exposure of Young Interventional Cardiologists. Am J Cardiol. 2022;181:25-31.

83. Patel AD, Gallagher AG, Nicholson WJ, Cates CU. Learning curves and reliability measures for virtual reality simulation in the performance assessment of carotid angiography. J Am Coll Cardiol. 2006;47(9):1796-802.

84. Li S, Cui J, Hao A, Zhang S, Zhao Q. Design and Evaluation of Personalized Percutaneous Coronary Intervention Surgery Simulation System. IEEE Trans Vis Comput Graph. 2021;27(11):4150-60.

85. Salavitabar A, Zampi JD, Thomas C, Zanaboni D, Les A, Lowery R, et al. Augmented Reality Visualization of 3D Rotational Angiography in Congenital Heart Disease: A Comparative Study to Standard Computer Visualization. Pediatr Cardiol. 2023.

86. Zhang R, Xu B, Dou K, Guan C, Zhao Y, Wang X, et al. Post-PCI outcomes predicted by pre-intervention simulation of residual quantitative flow ratio using augmented reality. Int J Cardiol. 2022;352:33-9.

87. Opolski MP, Debski A, Borucki BA, Staruch AD, Kepka C, Rokicki JK, et al. Feasibility and safety of augmented-reality glass for computed tomography-assisted percutaneous revascularization of coronary chronic total occlusion: A single center prospective pilot study. J Cardiovasc Comput Tomogr. 2017;11(6):489-96.

88. Alonso-Felipe M, Aguiar-Pérez JM, Pérez-Juárez M, Baladrón C, Peral-Oliveira J, Amat-Santos IJ. Application of Mixed Reality to Ultrasound-guided Femoral Arterial Cannulation During Real-time Practice in Cardiac Interventions. J Healthc Inform Res. 2023;7(4):527-41.

89. Bloom D, Colombo JN, Miller N, Southworth MK, Andrews C, Henry A, et al. Early preclinical experience of a mixed reality ultrasound system with active GUIDance for NEedle-based interventions: The GUIDE study. Cardiovasc Digit Health J. 2022;3(5):232-40.

90. Chahine J, Mascarenhas L, George SA, Bartos J, Yannopoulos D, Raveendran G, et al. Effects of a Mixed-Reality Headset on Procedural Outcomes in the Cardiac Catheterization Laboratory. Cardiovasc Revasc Med. 2022;45:3-8.

91. Salavitabar A, Whiteside W, Zampi JD. Feasibility of intraprocedural augmented reality visualisation of 3D rotational angiography in congenital cardiac catheterisation. Cardiol Young. 2023;33(3):476-8.

92. Witowski J, Darocha S, Kownacki Ł, Pietrasik A, Pietura R, Banaszkiewicz M, et al. Augmented reality and three-dimensional printing in percutaneous interventions on pulmonary arteries. Quant Imaging Med Surg. 2019;9(1):23-9.

93. Zablah JE, Rodriguez SA, Leahy R, Morgan GJ. Implementation of virtual reality for patient distraction during diagnostic cardiac catheterisation. Cardiol Young. 2022;32(2):323-7.

94. Higami H, Saito H, Endo H, Matsuo H, Tsuchikane E. A case report of virtual reality-guided percutaneous coronary intervention for anomalous origin of right coronary artery chronic total occlusion. Eur Heart J Case Rep. 2023;7(10):ytad507.

95. Sadeghi AH, Wahadat AR, Dereci A, Budde RPJ, Tanis W, Roos-Hesselink JW, et al. Remote multidisciplinary heart team meetings in immersive virtual reality: a first experience during the COVID-19 pandemic. BMJ Innov. 2021;7(2):311-5.

96. Roguin A, Beyar R. Real case virtual reality training prior to carotid artery stenting. Catheter Cardiovasc Interv. 2010;75(2):279-82.

97. Opolski MP, Debski A, Borucki BA, Szpak M, Staruch AD, Kepka C, et al. First-in-Man Computed Tomography-Guided Percutaneous Revascularization of Coronary Chronic Total Occlusion Using a Wearable Computer: Proof of Concept. Can J Cardiol. 2016;32(6):829.e11-3.

98. Goto J, Niizeki T, Iwayama T, Sasaki T, Higami H, Watanabe M. Virtual Reality-Guided Simulation for Percutaneous Coronary Intervention in a Patient with Anatomical Anomalies: A Case Report. Am J Case Rep. 2024;25:e944485.

99. Groninger H, Stewart D, Fisher JM, Tefera E, Cowgill J, Mete M. Virtual reality for pain management in advanced heart failure: A randomized controlled study. Palliat Med. 2021;35(10):2008-16.

100. Herbert VM, Perry RJ, LeBlanc CA, Haase KN, Corey RR, Giudice NA, et al. Developing a Smartphone App With Augmented Reality to Support Virtual Learning of Nursing Students on Heart Failure. Clinical Simulation in Nursing. 2021;54:77-85.

101. Jiravska Godula B, Jiravsky O, Matheislova G, Kuriskova V, Valkova A, Puskasova K, et al. Virtual Reality for Patient Education about Hypertension: A Randomized Pilot Study. J Cardiovasc Dev Dis. 2023;10(12).

102. Hessabi M, Sajjadi M, Shareinia H, Rouhani M. The Effect of Virtual Reality on Anxiety in Patients Admitted to the Cardiac Care Unit. International Journal of Pharmaceutical and Phytopharmacological Research. 2020;10(2):37-42.

103. Li YY, Peng J, Ping YY, Jia Jun W, Lu Y, Liu JJ, et al. Virtual reality-based cognitive-behavioural therapy for the treatment of anxiety in patients with acute myocardial infarction: a randomised clinical trial. Gen Psychiatr. 2024;37(2):e101434.

104. Behera SK, Punn R, Menendez M, Be C, Moon S, Zuniga M, et al. A Prospective Randomized Controlled Trial Using Virtual Reality in Pediatric Pre-intervention Echocardiograms to Decrease Child Anxiety and Fear. Pediatr Cardiol. 2024.

105. Choi S, Nah S, Cho YS, Moon I, Lee JW, Ah Lee C, et al. Accuracy of visual estimation of ejection fraction in patients with heart failure using augmented reality glasses. Heart. 2024;110(6):432-40.

106. Nas J, Thannhauser J, Vart P, van Geuns RJ, Muijsers HEC, Mol JQ, et al. Effect of Face-to-Face vs Virtual Reality Training on Cardiopulmonary Resuscitation Quality: A Randomized Clinical Trial. Jama Cardiology. 2020;5(3):328-35.

107. Bruining N, Roelandt J, Grunst G, Berlage T, Waldinger J, Mumm B, editors. Virtual heart model as an orientation tool in three-dimensional echocardiography: A feasibility study. 26th Annual Meeting on Computers in Cardiology; 1999 Sep 26-29; Hannover, Germany1999.

108. Nielsen MS, Clausen JH, Hoffmann-Petersen J, Konge L, Nielsen AB. Can virtual-reality simulation ensure transthoracic echocardiography skills before trainees examine patients? Int J Med Educ. 2022;13:267-73.

109. Forgiarini A, Deroma L, Buttussi F, Zangrando N, Licata S, Valent F, et al. Introducing Virtual Reality in a STEMI Coronary Syndrome Course: Qualitative Evaluation with Nurses and Doctors. Cyberpsychol Behav Soc Netw. 2024;27(6):387-98.

110. O'Sullivan DM, Foley R, Proctor K, Gallagher S, Deery A, Eidem BW, et al. The Use of Virtual Reality Echocardiography in Medical Education. Pediatr Cardiol. 2021;42(4):723-6.

111. Balian S, McGovern SK, Abella BS, Blewer AL, Leary M. Feasibility of an augmented reality cardiopulmonary resuscitation training system for health care providers. Heliyon. 2019;5(8):e02205.

112. Mehrpooya M, Mojtahedzadeh R, Sherafati A, Aalaa M, Mohammadi A. Comparative study of AR infographic posters vs. offline videos for micro-video delivery in cardiology education. J Vis Commun Med. 2024;47(1):1-7.

113. Hilt AD, Hierck BP, Eijkenduijn J, Wesselius FJ, Albayrak A, Melles M, et al. Development of a patient-oriented Hololens application to illustrate the function of medication after myocardial infarction. Eur Heart J Digit Health. 2021;2(3):511-20.

114. Hilt AD, Mamaqi Kapllani K, Hierck BP, Kemp AC, Albayrak A, Melles M, et al. Perspectives of Patients and Professionals on Information and Education After Myocardial Infarction With Insight for Mixed Reality Implementation: Cross-Sectional Interview Study. JMIR Hum Factors. 2020;7(2):e17147.

115. Galvez R, Wallon RC, Shackelford L, Amos JR, Rowen JL. Use of Virtual Reality to Educate Undergraduate Medical Students on Cardiac Peripheral and Collateral Circulation. Medical Science Educator. 2021;31(1):19-22.

116. Skalidis I, Arangalage D, Kachrimanidis I, Antiochos P, Tsioufis K, Fournier S, et al. Metaverse-based cardiac magnetic resonance imaging simulation application for overcoming claustrophobia: a preliminary feasibility trial. Future Cardiol. 2024;20(4):191-5.

117. Yogev D, Tejman-Yarden S, Feinberg O, Parmet Y, Goldberg T, Illouz S, et al. Proof of concept: Comparative accuracy of semiautomated VR modeling for volumetric analysis of the heart ventricles. Heliyon. 2022;8(11).

118. De Ponti R, Marazzi R, Ghiringhelli S, Salerno-Uriarte JA, Calkins H, Cheng A. Superiority of simulator-based training compared with conventional training methodologies in the performance of transseptal catheterization. J Am Coll Cardiol. 2011;58(4):359-63.

119. Chang SL, Kuo MJ, Lin YJ, Chen SA, Chen CT, Yang YY, et al. Virtual reality-based preprocedural education increases preparedness and satisfaction of patients about the catheter ablation of atrial fibrillation. J Chin Med Assoc. 2021;84(7):690-7.

120. Gorbaty B, Arango S, Buyck D, James RC, Porter ST, Iaizzo P, et al. Virtual Reality-based Methods for Training Novice Electrophysiology Trainees-A Pilot Study. J Innov Card Rhythm Manag. 2023;14(9):5583-99.

121. Hermans ANL, Betz K, Verhaert DVM, den Uijl DW, Clerx K, Debie L, et al. 360° Virtual reality to improve patient education and reduce anxiety towards atrial fibrillation ablation. Europace. 2023;25(3):855-62.

122. Coulibaly I, Cardelli LS, Duflos C, Moulis L, Mandoorah B, Nicoleau J, et al. Virtual Reality Hypnosis in the Electrophysiology Lab: When Human Treatments Are Better than Virtual Ones. J Clin Med. 2022;11(13).

123. Roxburgh T, Li A, Guenancia C, Pernollet P, Bouleti C, Alos B, et al. Virtual Reality for Sedation During Atrial Fibrillation Ablation in Clinical Practice: Observational Study. J Med Internet Res. 2021;23(5):e26349.

124. Chang SL, Kuo MJ, Lin YJ, Chen SA, Yang YY, Cheng HM, et al. Virtual reality informative aids increase residents' atrial fibrillation ablation procedures-related knowledge and patients' satisfaction. J Chin Med Assoc. 2021;84(1):25-32.

125. Balsam P, Borodzicz S, Malesa K, Puchta D, Tymińska A, Ozierański K, et al. OCULUS study: Virtual reality-based education in daily clinical practice. Cardiol J. 2019;26(3):260-4.

126. Southworth MK, Silva JNA, Blume WM, Van Hare GF, Dalal AS, Silva JR. Performance Evaluation of Mixed Reality Display for Guidance During Transcatheter Cardiac Mapping and Ablation. IEEE J Transl Eng Health Med. 2020;8:1900810.

127. Prakosa A, Southworth MK, Avari Silva JN, Silva JR, Trayanova NA. Impact of augmented-reality improvement in ablation catheter navigation as assessed by virtual-heart simulations of ventricular tachycardia ablation. Comput Biol Med. 2021;133:104366.

128. Silva JNA, Privitera MB, Southworth MK, Silva JR. Development and Human Factors Considerations for Extended Reality Applications in Medicine: The Enhanced ELectrophysiology Visualization and Interaction System (ĒLVIS). Virtual Augment Mixed Real (2020). 2020;12191:341-56.

129. Jang J, Tschabrunn CM, Barkagan M, Anter E, Menze B, Nezafat R. Three-dimensional holographic visualization of high-resolution myocardial scar on HoloLens. PLoS One. 2018;13(10):e0205188.

130. Serfözö PD, Sandkühler R, Blümke B, Matthisson E, Meier J, Odermatt J, et al. An augmented reality-based method to assess precordial electrocardiogram leads: a feasibility trial. Eur Heart J Digit Health. 2023;4(5):420-7.

131. James RC, Monsky WL, Jorgensen NW, Seslar SP. Virtual-Reality Guided Versus Fluoroscopy-Guided Transseptal Puncture in a Cardiac Phantom. Journal of Invasive Cardiology. 2020;32(2):76-81.

132. Bloom D, Catherall D, Miller N, Southworth MK, Glatz AC, Silva JR, et al. Use of a mixed reality system for navigational mapping during cardiac electrophysiological testing does not prolong case duration: A subanalysis from the Cardiac Augmented REality study. Cardiovasc Digit Health J. 2023;4(4):111-7.

133. Avari Silva JN, Southworth MK, Blume WM, Andrews C, Van Hare GF, Dalal AS, et al. First-In-Human Use of a Mixed Reality Display During Cardiac Ablation Procedures. JACC Clin Electrophysiol. 2020;6(8):1023-5.

134. Knecht S, Brantner P, Cattin P, Tobler D, Kühne M, Sticherling C. State-of-the-art multimodality approach to assist ablations in complex anatomies-From 3D printing to virtual reality. Pacing Clin Electrophysiol. 2019;42(1):101-3.

135. Lodziński PR, Balsam P, Peller M, Kamiński J, Opolski G. First-in-man percutaneous pulmonary vein isolation enhanced by augmented reality system. Kardiol Pol. 2018;76(2):475.

136. Rivero A, Torp K, Klingbeil R, Kusumoto F. Virtual Reality as an Anesthetic Alternative for Ablation of Noninducible Ventricular Arrhythmia. J Cardiothorac Vasc Anesth. 2024;38(5):1198-202.

137. Maytin M, Daily TP, Carillo RG. Virtual reality lead extraction as a method for training new physicians: a pilot study. Pacing Clin Electrophysiol. 2015;38(3):319-25.

138. Squara F, Bateau J, Scarlatti D, Bun SS, Moceri P, Ferrari E. Virtual Reality for the Management of Pain and Anxiety in Patients Undergoing Implantation of Pacemaker or Implantable Cardioverter Defibrillator: A Randomized Study. J Med Syst. 2024;48(1):28.

139. Drozdova A, Polokova K, Jiravsky O, Jiravska Godula B, Chovancik J, Ranic I, et al. Comparing Conventional Physician-Led Education with VR Education for Pacemaker Implantation: A Randomized Study. Healthcare (Basel). 2024;12(10).

140. Wong T, Darzi A, Foale RA, Schilling RJ, Ieee, Ieee, editors. A novel computerised virtual reality permanent: Pacing implantation simulator. 28th Annual Meeting on Computers in Cardiology; 2001 Sep 23-26; Rotterdam, Netherlands2001.

141. Mascheroni J, Mont L, Stockburger M, Patwala A, Retzlaff H, Gallagher AG. A validation study of intraoperative performance metrics for training novice cardiac resynchronization therapy implanters. Int J Cardiol. 2020;307:48-54.

142. Carretero EG, de Alarcón González A, de la Borbolla MG, Ciriza GG, Herrera V, Rueda EA, et al. Infections of cardiac implantable electronic devices and how to improve transvenous lead extraction by the virtual reality. Indian J Thorac Cardiovasc Surg. 2024;40(Suppl 1):138-49.

143. Witkowski M, Lodziński P, Zakrzewska-Koperska J, Krzyżanowski K, Zinka E, Sterliński M. Cardiac resynchronization device implantation supported by augmented reality visualization of computed tomography angiography reconstruction of the coronary sinus bed: the use of the Carna Life system. Kardiol Pol. 2019;77(4):484-5.

144. Opolski MP, Michałowska IM, Borucki BA, Nicińska B, Szumowski Ł, Sterliński M. Augmented-reality computed tomography-guided transcatheter pacemaker implantation in dextrocardia and congenitally corrected transposition of great arteries. Cardiol J. 2018;25(3):412-3.

145. Higaki A, Kawada Y, Hiasa G, Yamada T, Okayama H. Three-Dimensional Reconstruction of Pacemaker Lead Trajectory From Orthogonal Chest X-Rays: A Proof of Concept. Cureus. 2021;13(12):e20807.

146. Jóźwik S, Cieślik B, Gajda R, Szczepańska-Gieracha J. The Use of Virtual Therapy in Cardiac Rehabilitation of Female Patients with Heart Disease. Medicina (Kaunas). 2021;57(8).

147. Jóźwik S, Cieślik B, Gajda R, Szczepańska-Gieracha J. Evaluation of the Impact of Virtual Reality-Enhanced Cardiac Rehabilitation on Depressive and Anxiety Symptoms in Patients with Coronary Artery Disease: A Randomised Controlled Trial. J Clin Med. 2021;10(10).

148. Gulick V, Graves D, Ames S, Krishnamani PP. Effect of a Virtual Reality-Enhanced Exercise and Education Intervention on Patient Engagement and Learning in Cardiac Rehabilitation: Randomized Controlled Trial. J Med Internet Res. 2021;23(4):e23882.

149. Szczepańska-Gieracha J, Jóźwik S, Cieślik B, Mazurek J, Gajda R. Immersive Virtual Reality Therapy as a Support for Cardiac Rehabilitation: A Pilot Randomized-Controlled Trial. Cyberpsychol Behav Soc Netw. 2021;24(8):543-9.

150. García-Bravo S, Cano-de-la-Cuerda R, Domínguez-Paniagua J, Campuzano-Ruiz R, Barreñada-Copete E, López-Navas MJ, et al. Effects of Virtual Reality on Cardiac Rehabilitation Programs for Ischemic Heart Disease: A Randomized Pilot Clinical Trial. Int J Environ Res Public Health. 2020;17(22).

151. Maciołek J, Wąsek W, Kamiński B, Piotrowicz K, Krzesiński P. The impact of mobile virtual reality-enhanced relaxation training on anxiety levels in patients undergoing cardiac rehabilitation. Kardiol Pol. 2020;78(10):1032-4.

152. da Cruz MMA, Ricci-Vitor AL, Borges GLB, da Silva PF, Turri-Silva N, Takahashi C, et al. A Randomized, Controlled, Crossover Trial of Virtual Reality in Maintenance Cardiovascular Rehabilitation in a Low-Resource Setting: Impact on Adherence, Motivation, and Engagement. Phys Ther. 2021;101(5).

153. Vieira Á, Melo C, Machado J, Gabriel J. Virtual reality exercise on a home-based phase III cardiac rehabilitation program, effect on executive function, quality of life and depression, anxiety and stress: a randomized controlled trial. Disabil Rehabil Assist Technol. 2018;13(2):112-23.

154. Yuenyongchaiwat K, Boonkawee T, Pipatsart P, Tavonudomgit W, Sermsinsaithong N, Songsorn P, et al. Effects of virtual exercise on cardio-pulmonary performance and depression in cardiac rehabilitation phase I: A randomized control trial. Physiother Res Int. 2024;29(1):e2066.

155. Vieira ASD, de Melo M, Pinho A, Machado JP, Mendes JGM. The effect of virtual reality on a home-based cardiac rehabilitation program on body composition, lipid profile and eating patterns: A randomized controlled trial. European Journal of Integrative Medicine. 2017;9:69-78.

156. Wrzeciono A, Cieślik B, Kiper P, Szczepańska-Gieracha J, Gajda R. Exploratory analysis of the effectiveness of virtual reality in cardiovascular rehabilitation. Sci Rep. 2024;14(1):281.

157. Alves da Cruz MM, Ricci-Vitor AL, Bonini Borges GL, Fernanda da Silva P, Ribeiro F, Marques Vanderlei LC. Acute Hemodynamic Effects of Virtual Reality-Based Therapy in Patients of Cardiovascular Rehabilitation: A Cluster Randomized Crossover Trial. Arch Phys Med Rehabil. 2020;101(4):642-9.

158. Silva PF, Ricci-Vitor AL, Cruz MM, Borges GL, Garner DM, Marques Vanderlei LC. Comparison of acute response of cardiac autonomic modulation between virtual reality-based therapy and cardiovascular rehabilitation: a cluster-randomized crossover trial. Physiother Theory Pract. 2022;38(8):969-84.

159. Alves da Cruz M, Laurino M, Christofaro D, Ghisi G, Vanderlei L. Long-term effects of virtual reality-based therapy in cardiovascular rehabilitation: A longitudinal study. Physiother Theory Pract. 2022:1-9.

160. Vorwerg-Gall S, Perotti L, Dahms R, Stamm O, editors. Exergames as Synchronous Collaborative Remote Training in Older Adults with Hypertension: A Mixed Methods Pilot Study. 10th International Conference on Human Aspects of IT for the Aged Population (ITAP) Held as Part of the 26th International Conference on Human-Computer Interaction (HCII); 2024 Jun 29-Jul 04; Washington, DC2024.

161. Jóźwik S, Wrzeciono A, Cieślik B, Kiper P, Szczepańska-Gieracha J, Gajda R. The Use of Virtual Therapy in Cardiac Rehabilitation of Male Patients with Coronary Heart Disease: A Randomized Pilot Study. Healthcare (Basel). 2022;10(4).

162. da Cruz MMA, Grigoletto I, Ricci-Vitor AL, da Silva JM, Franco MR, Vanderlei LCM. Perceptions and preferences of patients with cardiac conditions to the inclusion of virtual reality-based therapy with conventional cardiovascular rehabilitation: A qualitative study. Braz J Phys Ther. 2022;26(3):100419.

163. Vieira Á, Gabriel J, Melo C, Machado J. Kinect system in home-based cardiovascular rehabilitation. Proc Inst Mech Eng H. 2017;231(1):40-7.

164. Klompstra LV, Jaarsma T, Strömberg A. AN IN-DEPTH, LONGITUDINAL EXAMINATION OF THE DAILY PHYSICAL ACTIVITY OF A PATIENT WITH HEART FAILURE USING A NINTENDO WII AT HOME: A CASE REPORT. Journal of Rehabilitation Medicine. 2013;45(6):599-602.
